# Distinct modes of interaction within eIF4F-like complexes and susceptibility to the RocA inhibitor for the ***Trypanosoma brucei*** EIF4AI translation initiation factor

**DOI:** 10.1371/journal.pone.0322812

**Published:** 2025-05-09

**Authors:** Danielle M. N. Moura, Amanda L. Soares, Adalúcia da Silva, João L. A. B. Ribeiro, Jack D. Sunter, Ludmila A. Assis, Mark Carrington, Osvaldo P. de Melo Neto

**Affiliations:** 1 Aggeu Magalhães Institute, Oswaldo Cruz Foundation (Fiocruz), Recife, Pernambuco, Brazil; 2 Department of Biological and Medical Sciences, Oxford Brookes University, Oxford, United Kingdom; 3 Department of Biochemistry - University of Cambridge, Cambridge, United Kingdom; University of Toronto, CANADA

## Abstract

Trypanosomatids are parasitic protozoa responsible for major human diseases which are characterized by unique gene expression mechanisms. mRNA translation in these parasites is associated with multiple eIF4F-like complexes, required for mRNA recruitment and ribosome binding. The eukaryotic eIF4F is generally known to require the action of eIF4A, an ATP-dependent RNA helicase, in order to function properly, but not all trypanosomatid eIF4F complexes might require EIF4AI, their single eIF4A homologue. In mammals, eIF4A is known to be targeted by specific inhibitors and can thus be considered a potential target for a selective inhibition of translation in these parasites. Here, aiming to better define the EIF4AI functionality, we started by investigating its interactome in *Trypanosoma brucei*, confirming a strong interaction with only one of five eIF4F-like complexes found in trypanosomatids, based on the EIF4E4/EIF4G3 subunits. Nevertheless, when the interactome of a mutant EIF4AI (DEAD/DQAD), known to be impacted on its ATPase activity, was investigated, the only eIF4F-like complex found was based on the EIF4E3/EIF4G4 pair, with many translation-related and other proteins also found with the mutant protein. When both wild-type and mutant proteins were also investigated through a fluorescent-based tethering assay, a stimulatory effect on mRNA expression was confirmed for EIF4AI, but not for the mutant protein. Sensitivity to the Rocaglamide A (RocA) inhibitor, which targets the mammalian eIF4A, was also investigated, with the inhibitor blocking the stimulation seen on the tethering assay. Parasite susceptibility to RocA was further assessed in *T. brucei* and *Leishmania infantum*, with both, and specially *T. brucei*, being much less susceptible to the drug than mammalian cells. This phenotype correlates with changes in EIF4AI within the RocA binding pocket where, in comparison with the mammalian eIF4A, a phenylalanine to valine substitution in the *T. brucei* EIF4AI likely impairs RocA binding. Our results help better define the EIF4AI mode of action in *T. brucei* and provide relevant data which might support future searches for specific EIF4AI inhibitors.

## Introduction

Trypanosomatids are parasitic protozoa which include several pathogenic species in the genera *Leishmania* and *Trypanosoma*. They are responsible for a range of diverse diseases of medical and veterinary importance with an impact on the economic development of several low-income populations. Specific and non-toxic drugs for the treatment of many of these infections are still lacking, despite the considerable number of studies devoted to the understanding of the basic biology of trypanosomatids and focusing on the search for novel therapeutic drugs [1,2]. Trypanosomatids separated very early from other eukaryotic lineages and are characterized by a large number of divergent molecular mechanisms, many of which still need to be better defined [3].

One unique feature of all trypanosomatids is the absence of gene-specific transcriptional control, with mRNA and protein levels set by post-transcriptional events including mRNA turnover and translation [4]. There is polycistronic transcription of protein coding genes by RNA polymerase II, followed by co-transcriptional processing into monocistronic transcripts through *trans*-splicing of a capped, 39 nucleotides long, mini-exon to the 5’ end of the mRNAs. All mature mRNAs then have a common 5’ end, consisting of the capped Spliced-Leader (SL) sequence, and which precedes the typical 5’ untranslated regions (5’ UTRs) of the different mRNAs. The 5’ cap consists of 7-methyl-GTP, generally found in most eukaryotic mRNAs, followed by four methylated nucleotides and which form a modified cap structure, named cap4 [5].

The eukaryotic cap is required at the start of translation for the recognition of the mRNA by the small (40S) ribosomal subunit, a step which precedes the 40S binding to the mRNA and subsequent scanning of the 5’ UTR in order to find the translation initiation codon, AUG. Cap recognition is mediated by the translation initiation factor eIF4F, formed through the binding of the eIF4E subunit, the cap binding protein, to the multidomain/scaffold protein eIF4G, which is known to mediate the eIF4F interactions with a number of protein partners, including multiple subunits of the large, ribosome bound, eIF3 complex [6–9]. A third eIF4F subunit in mammals is the RNA helicase eIF4A, responsible for the eIF4F unwinding activity which removes secondary structures in the mRNA 5’ UTR, which might prevent 40S subunit binding or its scanning in search of the AUG. This unwinding/helicase activity can be reinforced by a second RNA helicase, DDX3, as well as other initiation factors, eIF4B and eIF4H, which might be required for mRNAs with more structured 5’ UTRs [8–13]. Both eIF4F formation and the eIF4A helicase activity have been investigated as potential targets for inhibitors which could be used to specifically inhibit mRNA translation in cancer cells [13–16] with eIF4A inhibitors also investigated for antiviral activity [17,18].

eIF4A is the prototype for the DEAD-box family of ATP-dependent RNA helicases, enzymes required in different events associated with the metabolism of RNAs. Members of this family have a conserved core containing the RNA binding and ATPase sites required to promote the helicase activity. The helicase core consists of two RecA domains separated by a linker region and forming a dumbbell-shaped structure, defined by the presence of nine typical conserved motifs [8,10,19,20]. Six of those motifs (Q, I, Ia, Ib, II and III) are found within the N-terminal domain whilst the remaining three (IV, V and VI) are mapped to the C-terminal domain. The DEAD-box motif (aspartate, glutamate, alanine and aspartate), part of the typical motif II, is involved in ATP binding and hydrolysis [21,22]. Three eIF4A homologues were originally described in mammals (eIF4AI, eIF4AII and eIF4AIII) [20,23], where eIF4AI and eIF4AII were found to be cytoplasmic proteins functionally active during translation [8], whilst eIF4AIII functions within the nucleus and participates in mRNA splicing and rRNA biogenesis [24,25]. Within eIF4F, the specific interaction between eIF4A and eIF4G requires multiple motifs in eIF4A which primarily binds to the central MIF4G domain of eIF4G, a conserved domain in all known eIF4G sequences [26,27]. A second eIF4A interacting region within eIF4G is mapped to the MA3 domain, localized within the eIF4G C-terminal region. The MA3 domain is missing from the *Saccharomyces cerevisiae* eIF4G and its absence might explain the low affinity of the interaction between the yeast eIF4G and eIF4A, with eIF4A not being considered a true eIF4F subunit in this microorganism [8,28].

A single eIF4A homologue active in translation, EIF4AI, is present in trypanosomatids [29]. In contrast, there are six eIF4E and five eIF4G homologues, forming a minimum of five different eIF4F-like complexes which may or may not include EIF4AI. Two of these eIF4F-like complexes have been shown to be most active in translation and form through the association of two distinct sets of eIF4E and eIF4G subunits, EIF4E4 with EIF4G3 and EIF4E3 with EIF4G4 [30]. EIF4G3 and EIF4G4 are characterized by short N-terminal regions, which nevertheless include eIF4E binding motifs, followed by the MIF4G domain and conserved C-terminal regions which might include MA3-like domains. Both full-length EIF4G3 as well as its MIF4G domain were found to bind efficiently to EIF4AI *in vitro*. In contrast, only the full-length EIF4G4 bound to EIF4AI in the same assay [31]. Recent studies, in both *Trypanosoma brucei* and *Leishmania infantum*, have confirmed the co-precipitation of EIF4AI with the native EIF4E4/EIF4G3 but not with EIF4E3/EIF4G4, the latter complex found to be preferentially enriched with other DEAD-box RNA helicases, such as HEL67, a Ded1 homologue. Co-precipitation experiments also revealed that the EIF4E4/EIF4G3 complex binds preferentially to mRNAs encoding ribosomal proteins, and which are characterized by very short 5’UTRs and are unlikely to have complex secondary structures. In contrast, the EIF4E3/EIF4G4 complex associated with a much wider range of mRNAs, encoding functionally unrelated proteins but excluding those encoding ribosomal proteins. These transcripts generally have 5’UTRs significantly longer than those found with EIF4E4/EIF4G3 [32,33].

It was once thought that the eIF4A activity would be mostly required for the translation of mRNAs having secondary structures within their 5’UTRs and which would otherwise impact on the 40S ribosomal binding or scanning [34,35]. This is at odds with the clear EIF4AI association with EIF4E4/EIF4G3 and the ribosomal protein mRNAs [33], a unique class of cellular mRNAs which in trypanosomatids have very short and presumably unstructured 5’UTRs [36]. However, recent experiments in other organisms have implied a more direct role for eIF4A function during ribosome recruitment which is independent of eIF4G but requires the eIF4A ATPase activity [37–42]. Here, considering that an eventual targeting of EIF4AI with specific helicase inhibitors in trypanosomatids requires a better definition of its mode of action during translation, we opted to investigate it further in *T. brucei*. We started by searching for co-precipitating proteins and investigated its function in a new fluorescence-based tethering assay. We evaluated both the wild-type and a mutant EIF4AI having a modified DEAD motif and also investigated the effect of the helicase inhibitor Rocaglamide A (RocA) on the EIF4AI function. Our results reinforce the association between EIF4AI and the EIF4E4/EIF4G3 based complex whilst also supporting a potential association with the EIF4E3/EIF4G4 pair, but no evidence was found for the EIF4AI association with the other eIF4F-like complexes found in trypanosomatids. The stimulatory effect by EIF4AI in a tethering assay was confirmed and found to be dependent on the integrity of the DEAD motif as well as sensitive to RocA. The lower sensitivity to this drug by different trypanosomatid species, however, was found to be associated with changes in the RocA binding site which directly impact on its activity. It implies important differences in the interaction between the drug and its EIF4AI target which should be relevant for future studies investigating related substances of therapeutic potential.

## Materials and methods

### Cloning procedures

Coding sequences for the wild-type (WT) and DQAD EIF4AI mutant were previously available [29] and were here subcloned between the *Hind*III and *BamH*I sites of the p2477 plasmid [43] to generate the constructs coding for tagged proteins having six copies of the HA epitope at their C-terminuses. For the tethering assays, the same fragments were likewise subcloned into a modified version of the p3927 plasmid, having a DNA segment coding for a N-terminal 44 amino acid sequence consisting of the λ N-22 peptide followed by the TY epitope, as previously described [31]. Also, a monocistronic reporter based on the expression of the fluorescent protein eGFP was generated. For that, the eGFP coding sequence was amplified by PCR and cloned between the *Hind*III and *BamH*I sites of the plasmid p3605 [44], where the resistance mark was replaced from blasticidin (bsr^R^) to neomycin (neo^R^). A synthetic sequence containing a single BoxB hairpin (5’- gat ctg tct cgt tac tgc cat aaa ata ATT GTA ATA TTC GGG CCC TGA AGA AGG GCC CTA TAT GCA AA cac cgg gtt gtg tgg cca aat ttg ttc ctc gag taa gga tcc-3’) was then subcloned downstream of the eGFP coding sequence, within the *BamH*I site, with the correct insertion and orientation confirmed by DNA sequencing (plasmid p4213). The construct without the sequence containing the BoxB hairpin was used as negative control (plasmid p4212).

### Parasite growth and transfection

Procyclic *T. brucei* cells, Lister 427, were cultivated at 27°C in SDM-79 supplemented with haemin, 10% heat-inactivated foetal calf serum (FCS) and 1% penicillin-streptomycin solution. Cultures were grown to mid-log phase for harvesting and production of total protein extract as previously described [45]. Transfection procedures were performed using standard conditions. For the p2477-derived plasmids (HA tag), the procyclic cell line Lister 427 29–13 was used [46]. For the tethering assay, plasmids p4212 and p4213 were transfected into a cell line previously generated with the p4106 plasmid, derived from pSMOX [47] and constitutively expressing the tetracycline repressor only. Transfected cells were selected in the presence of phleomycin (2.5 μg/mL), G418 (15 μg/mL) or blasticidin (10 μg/mL), according to the plasmid used to generate the corresponding cell lines. Expression of HA- and TY-tagged proteins was induced after tetracycline addition (1 μg/mL) and cellular growth was monitored by cell counting at regular intervals. Promastigotes of *Leishmania infantum* strain MHOM/MA/67/ITMAP-263 were cultured at 25°C, in Schneider’s insect medium pH 7.2 supplemented with 1% penicillin-streptomycin solution, 10% heat-inactivated FCS and haemin.

### Immunoprecipitation and mass spectrometry

Immunoprecipitations (IPs) were performed with *T. brucei* cytoplasmic extracts produced from log phase procyclic cells which expressed the HA-tagged proteins for 24h and were then ressuspended in lysis buffer (20 mM HEPES–KOH, pH7.4, 75 mM potassium acetate, 4 mM magnesium acetate, 2 mM DTT), supplemented with EDTA-free protease inhibitor cocktail (Roche), followed by lysis through cavitation at 70 Bar, as described previously [32]. The IPs were performed with magnetic beads (Pierce anti-HA magnetic beads - Thermo Scientific) and for the negative control the same beads were incubated with cytoplasmic extracts from non-transfected cells (from the 29–13 cell line). All the experiments were performed in triplicates, with the IP products eluted in sample buffer for SDS-PAGE and briefly migrated by SDS-PAGE. Gel fragments containing the immunoprecipitated polypeptides were then excised and sent for mass spectrometry analyses, after protein elution and trypsin digestion, in a LTQ Orbitrap XL EDT Thermo Scientific. These experiments were performed simultaneously for both native and mutant EIF4AI, with the same negative control for both. After peptide identification, only proteins found with a minimum of two peptides for at least two of the triplicates for either of the tagged baits were considered for subsequent analysis. Normalized intensity values for each co-precipitated protein were calculated for the whole set of IPs, as previously described [33], with average values for either of the tagged baits or the control then used to calculate the intensity enrichment ratios for the two baits in comparison with the control samples. All proteins with enrichment ratios greater than 1.5 are listed in the S1 and S2 Tables, with a selection of those shown in Tables 1 and 2.

**Table 1:** Selected enriched proteins co-precipitated with the HA-tagged EIF4AI_WT_ in *Trypanosoma brucei* procyclic cells.

TriTrypDB ID	Name	Protein description	Intensity	Enrichment
		**BAIT**		
Tb927.9.4680	EIF4AI	Eukaryotic Translation Initiation Factor 4A	4.23E + 09	8.3
		**eIF4F SUBUNITS**		
Tb927.6.1870	EIF4E4	Eukaryotic translation initiation factor 4E-4	1.19E + 07	31.7
Tb927.8.4820	EIF4G3	Eukaryotic translation initiation factor 4 gamma 3	3.99E + 06	7.4
		**OTHER TRANSLATION-RELATED PROTEINS**		
Tb927.10.13360	EEFTU	Elongation factor Tu, mitochondrial	4.39E + 07	1.72
Tb927.10.5840	EEF1β	Translation elongation factor 1-beta, putative	2.73E + 07	1.65
Tb927.11.8880	Sua5/YrdC	tRNA threonylcarbamoyl adenosine modification protein	2.35E + 07	1.82
Tb927.11.14120	PheRS	Phenylalanyl-tRNA synthetase alpha chain	2.17E + 07	2.18
		**RNA BINDING PROTEINS**		
Tb927.11.13280	gBP25^a^	Guide RNA binding protein 25. MRP2.	1.61E + 07	2.61
Tb927.7.2570	GRBC1^a^	Guide RNA associated protein, GAP2	7.68E + 06	∞
Tb927.2.5240	PRP19^b^	pre-mRNA splicing factor 19	3.71E + 06	1.91

Only translation related and RNA binding proteins that co-immunoprecipitated with the EIF4A_WT_-HA bait are shown. The full list of enriched proteins is shown in the S1 Table.

^a^Proteins which mainly localize to the mitochondria, based on the TrypTag database [49].

^b^Proteins which mainly localize to the nucleus, based on the TrypTag database [49].

**Table 2:** Selected enriched proteins co-precipitated with the HA-tagged EIF4AI_DQAD_ in *Trypanosoma brucei* procyclic cells.

TriTrypDB ID	Name	Protein description	Intensity	Enrichment
		**BAIT**		
Tb927.9.4680	EIF4AI	Eukaryotic Translation Initiation Factor 4A	1.53E + 09	3.04
		**eIF4F SUBUNITS**		
Tb927.11.10560	EIF4G4	Eukaryotic translation initiation factor 4 gamma 4	3.51E + 07	2.65
Tb927.11.11770	EIF4E3	Eukaryotic translation initiation factor 4E-3	1.09E + 07	1.52
		**OTHER TRANSLATION-RELATED PROTEINS**		
Tb927.10.5840	EEF1β	Translation elongation factor 1-beta, putative	2.94E + 07	1.78
Tb927.11.8880	Sua5/YrdC	tRNA threonylcarbamoyl adenosine modification protein	5.10E + 07	3.95
Tb927.11.14120	PheRS	Phenylalanyl-tRNA synthetase alpha chain	2.98E + 07	3.00
Tb927.11.15420	EIF3K	Eukaryotic translation initiation factor 3 subunit k	9.88E + 06	1.73
Tb927.9.5150	RPS6	Ribosomal protein S6, putative	5.35E + 06	22.66
		**RNA BINDING PROTEINS**		
Tb927.10.14550	HEL67	ATP-dependent RNA helicase.	5.65E + 07	3.92
Tb927.11.13280	gBP25 ^a^	Guide RNA binding protein 25. MRP2.	4.06E + 07	6.60
Tb927.10.540	SUB2 ^b^	ATP-dependent RNA helicase SUB2	3.28E + 07	2.04
Tb927.11.1710	gBP21 ^a^	Guide RNA-binding protein of 21 kDa. MRP1	3.13E + 07	6.68
Tb927.11.16550	ZC3H46	Zinc finger protein, C3H1 type-like	2.06E + 07	∞
Tb927.10.11760	PUF6	Pumilio/PUF RNA binding protein 6	1.12E + 07	2.65
Tb927.11.4450	ALBA2	ALBA domain protein	9.97E + 06	3.60
Tb927.11.510; Tb927.11.500	UBP1/2	RNA-binding protein, UBP1/UBP2	8.10E + 06	6.49
Tb927.10.13720	RBP29	RNA-binding protein 29, putative	7.82E + 06	2.50
Tb927.11.8870	MHEL61 ^a^	Mitochondrial DEAD-box RNA helicase	7.14E + 06	5.12
Tb927.11.1980	ZC3H41	Zinc finger protein family member, putative	7.01E + 06	2.70
Tb927.4.2040; Tb927.4.2030	ALBA3/4	DNA/RNA-binding protein Alba 3/Alba 4	6.65E + 06	34.27
Tb927.2.5240	PRP19 ^b^	Pre-mRNA splicing factor 19	5,98E + 06	3.09
Tb927.9.8740	DRBD3	Double RNA binding domain protein 3	5,90E + 06	11.79
Tb927.6.4440	RBP42	RNA-binding protein 42	5,36E + 06	10.71

Only translation related and RNA binding proteins that co-immunoprecipitated with the EIF4A_DQAD_-HA bait with average intensities greater than 5x10^6^ are shown. The full list of enriched proteins is shown in the S2 Table.

^a^Proteins which mainly localize to the mitochondria, based on the TrypTag database [49].

^b^Proteins which mainly localize to the nucleus, based on the TrypTag database [49].

To directly evaluate the differences in co-precipitated proteins found with the native and mutant EIF4AI, the raw mass-spectrometry data was also analysed independently with the Perseus software, version 2.1.3.0 [48] which excluded potential contaminants, reverse sequences, and proteins identified only by site during data processing. Protein intensities were then transformed by the software to a log_2_ scale, and missing values were imputed based on a normal distribution centred around the detection limit of the mass spectrometer. Statistical analysis was performed in Perseus using the Student’s t-test, comparing protein intensities between the EIF4AI-DQAD and EIF4AI-WT baits (both sets of triplicates). The results were visualized through a volcano plot, where the x-axis represents the differences in log_2_ intensities between the two baits and the y-axis displays the -Log(p) values, indicating the statistical significance of the observed differences. The significance threshold was defined based on the false discovery rate (FDR = 0.05) and an s0 parameter of 0.1. Individual values for all proteins shown in the volcano plot are listed in the S3 Table.

### Tethering assay

For this assay, a GFP-reporter procyclic cell line, named 4213, that constitutively expresses GFP mRNA containing one boxB stem loop RNA sequence within its 3’-UTR was generated (GFP + , BoxB+) and subsequently transfected with plasmids encoding either EIF4AI_WT_ or the EIF4AI_DQAD_ mutant, both having the N-terminal λN-peptide/TY epitope fusion, in a tetracycline inducible system. After tetracycline addition (+TET), clones expressing the λN-TY-EIF4AI_WT_ or λN-TY-EIF4AI_DQAD_ proteins were analysed for eGFP protein expression at 48h post-induction, on average 2 x 10^4^ cells per experiment, in a FACSCalibur system (BD Biosciences). Non-induced cells (-TET) as well as cells from the non-transfected 427 cell line (GFP negative) and the original GFP-expressing 4213 cell line (GFP positive) were used as controls. As a control for the tethering assay, parallel experiments were carried out with another reporter cell line that expresses the same eGFP mRNA but without the BoxB sequence (cell line 4212; GFP + , BoxB-). As another control for the experiment, the EIF4E1 CDS was also cloned into the modified p3927 plasmid and tested in this system.

The analyses were done in triplicates in two independent experiments. Another set of experiments were performed using the RocA inhibitor at 1 µ M. For these experiments, the cell lines were initially induced with tetracycline for 24h and then incubated in presence (+RocA) or absence (-RocA) for the subsequent 24 hours, when the GFP expression was then evaluated by flow cytometry as described. All results were analysed using the FlowJo software version 10.10.0. Statistical analyses were performed with t-test and ANOVA on the GraphPad Prism v.8 software.

### Inhibition with Rocaglamide A (RocA)

To test the inhibitory effect of RocA, *T. brucei* procyclic cells were cultured in SDM-79 medium to a density of 1x10^6^ cells/mL in a 48-well plate. Five different concentrations of RocA (Sigma-Aldrich) were then established for the IC_50_ determination (10 µ M to 0.625 µ M dissolved in dimethylsufoxide (DMSO)), with cell-growth monitored after 48 hours of exposure to the inhibitor. As a control, a culture without inhibitor was used, but with the diluent (1% DMSO). The growth profile of cultures was evaluated in triplicate, in at least two independent experiments, by counting in a Neubauer chamber at 48h. Likewise, *L. infantum* promastigotes were cultured and diluted in Schneider’s Insect Medium to a density of 1x10^6^ cells/mL in a 48-well plate. The RocA concentrations established were 10-fold less than for *T. brucei* (1 µ M to 0.0625 µ M). The half-maximal inhibitory concentration (IC_50_) was calculated using non-linear regression in GraphPad Prism v.8 software.

### *In silico* analysis

Protein sequences were retrieved from the Uniprot database (https://www.uniprot.org/) with the following accession numbers: *Tb*EIF4AI (Q38F76), *Tc*EIF4AI (Q4E162), *Li*EIF4AI (A0A381M920), *Sce*IF4AI (P10081), and *Hs*EIF4AI (P60842). The sequence alignments were performed using the MAFFT software, version 42.0, found at Unipro UGENE (Integrated Bioinformatics Tools). The *Tb*EIF4AI and *Li*EIF4AI protein sequences were modelled with the AlphaFold2 structure prediction tool (https://alphafold.ebi.ac.uk/), powered by deep learning neural network algorithms from Google DeepMind (https://deepmind.com/). The EIF4AI:RocA:RNA interaction models were generated from the superposition of the modelled proteins and the resolved structure of the human eIF4AI in complex with RocA and polypurine RNA, available at the RCSB Protein Data Bank (https://www.rcsb.org/) (PDB ID: 5ZC9), using PyMol (version 3.7) to position the RNA and the RocA ligands in the models generated with AlphaFold2. These models were subjected to a force field to minimize energy in YASARA, and the interactions were evaluated using Discovery Studio 2021. The quality of the structural models was evaluated using the Ramachandran plot (https://saves.mbi.ucla.edu/).

## Results

### Defining the *T. brucei* EIF4AI interactome

In previous work, the EIF4AI association with the EIF4E3/EIF4G4 and EIF4E4/EIF4G3 based complexes from *T. brucei* was investigated through assays using as baits the individual eIF4E (EIF4E3 and EIF4E4) or eIF4G (EIF4G3 and EIF4G4) subunits. These experiments were carried out after modifying the endogenous loci coding for these various proteins and expressing them as fusions with the enhanced yellow fluorescent protein (EYFP), used as a tag for immunoprecipitation assays [33]. Here, in order to better define the EIF4AI interactions with these and other eIF4F-like complexes from *T. brucei,* we first set out to perform a reciprocal immunoprecipitation assay using as bait a tagged EIF4AI. For this experiment we opted to use a tetracycline inducible EIF4AI transgene [29] but replacing the myc tag with six copies of the HA (hemagglutinin) epitope. Expression of the HA-tagged protein was first evaluated after tetracycline induction of different clones through western blots using a monoclonal anti-HA antibody and protein extracts from the transgenic strains, non-induced and induced for 24 hours (S1 Fig).

Cytoplasmic lysates derived from exponentially grown cells from the transgenic cell lines expressing the HA-tagged EIF4AI were next used in immunoprecipitation assays using anti-HA coupled beads, in experiments performed in parallel with extracts from the parental cell lines, as negative controls. The precipitated samples, in triplicates, were then evaluated through western blots to confirm the efficiency of the procedure (also shown in S1 Fig) and sent to mass spectrometry analysis for the identification of the co-precipitated proteins. Normalized intensities from each individual protein co-precipitated with the tagged EIF4AI (EIF4AI_WT_-HA) were then used to calculate enrichment ratios in comparison with the negative control. The complete list of proteins enriched 1.5-fold or more with EIF4AI_WT_-HA in comparison with the control is included in the S1 Table. A subset of those, generally the ones most enriched or judged to be more functionally relevant, are compiled within Table 1.

As expected, EIF4AI was the protein with the highest intensity among those within the inclusion criteria which co-precipitated with the anti-HA beads. Nevertheless, it was also the topmost protein found in the negative control samples possibly as a consequence of its substantial abundance, previously assessed through direct quantitation using specific antibodies [29] and more recently confirmed in a large-scale proteomic analysis of *T. brucei* proteins [50]. This might explain the only moderate enrichment ratio seen for the tagged EIF4AI when samples from cells expressing the HA-tagged protein were compared with those from control cells (~8-fold enrichment). Indeed, few proteins associated with translation were found among those enriched in the immunoprecipitations, but significant exceptions were EIF4E4 and EIF4G3, both found with enrichments similar to (EIF4G3) or greater than (EIF4E4) the tagged EIF4AI. No other eIF4E or eIF4G homologue was found among the enriched proteins. In all, only four other proteins related to translation, the elongation factor 1-beta (EEF1β) and elongation factor Tu (EEFTU), as well as two other associated with the tRNA metabolism (Sua5/YrdC and PheRS), were found in this list, but with low enrichment values. Neither ribosomal proteins nor other RNA helicases were enriched. In contrast, a large number of metabolic enzymes and cytoskeletal and flagellar proteins were found enriched with EIF4AI, some with very substantial intensities and enrichment values (S1 Table). Overall, our results reinforce the strong and specific association between EIF4AI and the EIF4E4/EIF4G3 based complex, as previously indicated by the immunoprecipitations experiments carried out using these eIF4E/eIF4G subunits as baits [33].

### Interactome of an EIF4AI DEAD-box mutant

Early studies identified a glutamate to glutamine mutation which, when introduced into the DEAD motif of the mammalian eIF4A (modified to DQAD), inactivates the ATPase and RNA helicase activities and leads to a strong dominant negative effect on translation when the mutant protein was tested using *in vitro* translation assays [51]. An identical mutation introduced into the myc-tagged, tetracycline inducible, *T. brucei* EIF4AI caused a slow growth phenotype [29]. Here, to investigate changes in co-precipitated proteins induced by the DQAD mutation, we first generated cell lines expressing the HA-tagged version of the mutant protein which was also expressed in a tetracycline inducible form (S1 Fig). As previously seen for the myc-tagged EIF4AI, expression of the HA-tagged mutant protein (EIF4AI_DQAD_-HA) led to a slight reduction in the rate of cell proliferation (~15% inhibition) when compared with the control cell line (29–13), not seen after expression of the native EIF4AI. Immunoprecipitation assays, equivalent to those carried out with the native protein, were then performed in triplicates and the samples also analysed by mass spectrometry, as described above.

The full list of proteins co-precipitated with the EIF4AI_DQAD_-HA mutant enriched 1.5-fold or more, in comparison with the negative control is shown in the S2 Table. Overall enrichment for the tagged bait was lower for the mutant protein when compared to the wild-type EIF4AI (3-fold enrichment for EIF4AI_DQAD_-HA/ ~ 8-fold enrichment for EIF4AI_WT_-HA), contrasting with a much larger number of proteins enriched with the EIF4AI_DQAD_-HA (131 for EIF4AI_DQAD_-HA/ 71 for EIF4AI_WT_-HA) and with substantial changes in the profile of enriched proteins. Noteworthy are the changes in translation associated factors found enriched with the mutant EIF4AI, in particular the absence of the EIF4E4/EIF4G3 pair. In contrast, a second set of eIF4F subunits, EIF4E3 and EIF4G4, were found enriched with the mutant EIF4AI, although with low enrichment values (see also in Table 2). Other translation-related proteins specifically enriched with the mutant EIF4AI were the EIF3K subunit of the eIF3 complex and the S6 ribosomal protein, a component of the 40S ribosomal subunit, both proteins which, in humans, have been shown to interact with eIF4A or localizes near to it [40].

When the overall intensities are considered, some of the most-enriched proteins found associated with the mutant EIF4AI are proteins associated with proteolytic pathways as well as chaperones and chaperonin subunits, with only a limited number of those also found with the EIF4AI_WT_-HA (all listed in the S2 Table). Many metabolic enzymes are also found among the top-most proteins co-precipitated with the mutant EIF4AI, with only a few of those coinciding with the ones enriched with the wild-type bait. Especially relevant, however, is the large number of proteins associated with the metabolism of mRNAs specifically enriched with the mutant bait, such as several RNA helicases (HEL67, SUB2 and MHEL61/REH1) and distinct sets of proteins having RNA binding domains, as highlighted in Table 2. Some of these have been shown to localize preferentially to the mitochondria or the nucleus, as indicated, and the reasons for their co-precipitation with the bait are unclear. Nevertheless, many of the RNA binding proteins found with the bait have been shown to either stimulate or decrease the expression of a reporter mRNA in previously published tethering assays, therefore classified as post-transcriptional activators or repressors [52,53]. Also found particularly enriched with EIF4AI_DQAD_-HA, and listed in the S2 Table only, are several subunits of the CCR4-NOT complex and which have also been shown previously to be post-transcriptional repressors (CAF1, NOT2, NOT5, NOT10 and NOT11), with only one of those (NOT11) also found with the EIF4AI_WT_-HA, but with a lower enrichment. This remarkably distinct set of proteins found co-precipitating with the mutant EIF4AI indicates differences in interacting properties which might nevertheless be of functional relevance and might provide clues as to the EIF4AI mode of action in association with different eIF4F-like complexes.

### Impact of the DEAD/DQAD mutation on the EIF4AI interactions

**Fig 1:**
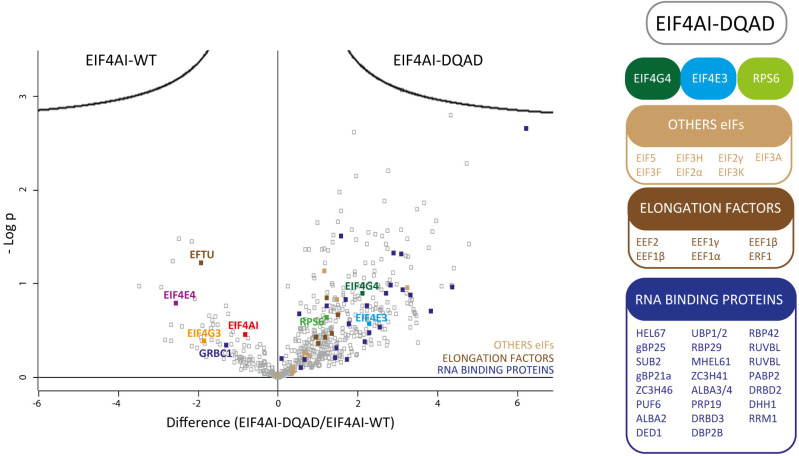
Comparative analysis of proteins differentially co-precipitated with the wild-type and mutant EIF4AI. The volcano plot compares the protein interaction profiles obtained for the EIF4AI_WT_-HA and EIF4AI_DQAD_-HA baits. The x-axis represents the differences in log_2_-transformed protein intensities between EIF4AI_WT_-HA and EIF4AI_DQAD_-HA, while the y-axis shows the -log(p) values, reflecting the statistical significance of the observed differences. Proteins with significant changes are highlighted/annotated, including key RNA-binding proteins, elongation factors, and other translation-related factors. A schematic categorization of selected proteins enriched in the EIF4AI-DQAD condition is shown on the right, with these proteins grouped into relevant functional classes: eIF4F subunits (EIF4G4, EIF4E3), RPS6 (ribosomal protein S6), other eukaryotic initiation factors (EIF5 and subunits of both eIF3 and eIF2 complexes); elongation factors (e.g., EEF2, EEF1α) and RNA-binding proteins.

The altered set of interaction partners found with the DQAD EIF4AI mutant is relevant, considering that the DEAD motif does not directly participate in known protein-protein interactions. This motif is positioned internally within the N-terminal domain of eIF4A homologues, facing the ATP binding pocket and unlikely to be accessible to other proteins, with the interaction with eIF4G being mostly mediated by the eIF4A C-terminal domain [26,27]. To better evaluate the differences between the wild-type EIF4AI and the DQAD mutant on likely protein partners, we directly compared changes in association between the co-precipitated proteins and the tagged baits. For this comparison we reassessed the original mass spectrometry data and used it to build the Volcano plot shown in Fig 1, plotting differences in log_2_-transformed protein intensities between the EIF4AI_WT_-HA and EIF4AI_DQAD_-HA baits with the -log(p) values derived from the statistical analysis of these differences. Selected proteins, arbitrarily judged to be more relevant, are indicated in the figure, with the complete set of identified proteins, plus the corresponding differences and -log(p) values, listed in the S3 Table. This comparative analysis highlights the association of the native protein with the EIF4E4/EIF4G3 complex, while both the EIF4E3/EIF4G4 subunits are preferentially found with the mutant EIF4AI. More relevant, however, was the large number of other translation related proteins which were seen more associated with the mutant protein, such as ribosomal proteins, aminoacyl tRNA synthetases and translation factors, including multiple subunits of the eIF3 and eIF2 complexes as well as EIF5 (Fig 1). Other RNA-related proteins previously shown to be specifically related to the EIF4E3/EIF4G4 complex [33] were also seen to have an increased association with the mutant EIF4AI, such as PABP2, the RNA binding proteins DRBD2 and RRM2 and the RNA helicase DBP2B. In contrast, PABP1 and the RNA binding protein RBP23, known partners of the EIF4E4/EIF4G3 complex, were not found amongst those associated with the wild-type EIF4AI. Other proteins increased with the mutant EIF4AI included a large number of proteasomal subunits and proteins associated with degradation pathways, many not seen in the previous analysis, as well as more chaperones and chaperonin subunits. In conclusion, these results reinforce the association of the mutant protein with the EIF4E3/EIF4G4 complex and its co-precipitation with ribosomes and translation factors, contrasting with what is observed for the wild-type EIF4AI.

### *T. brucei* EIF4AI stimulates expression of a reporter mRNA, dependent on the integrity of its DEAD motif

**Fig 2:**
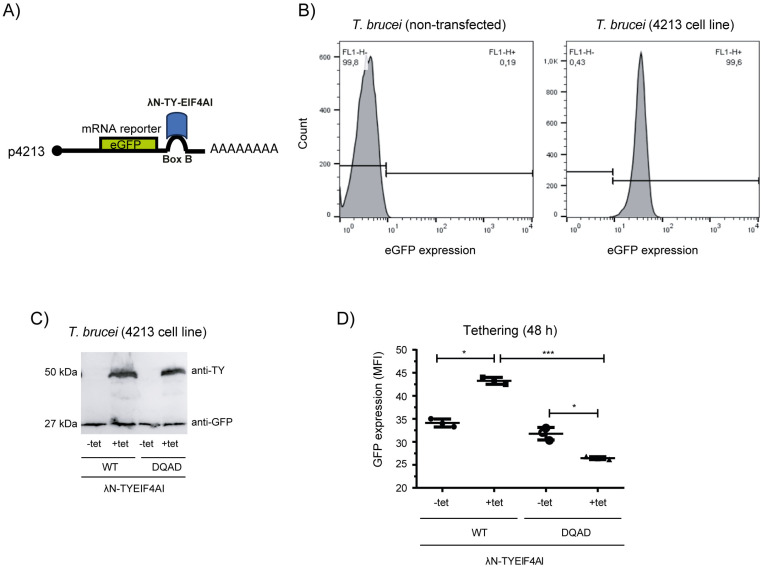
Effect of the tethering of EIF4AI on the expression of a reporter mRNA. A) Schematic representation of the mRNA encoding the eGFP reporter with an added boxB motif, represented by a stem loop, placed within its 3’UTR, and the binding of the λN-TY-EIF4AI. B) Histogram representing the eGFP expression detected by flow cytometry in non-transfected *T. brucei* procyclic cells and in the transgenic 4213 cell line. C) Western blot probed with the anti-TY and anti-GFP monoclonal antibodies and confirming the expression of both the *λ*N-TY- EIF4AI_WT_ and *λ*N-TY-EIF4AI_DQAD_ in representative transgenic clones induced (+tet) or not induced (-tet) with tetracycline for 24 hours. D) Quantitative analysis of eGFP expression 48 hours after tetracycline induction. The results are derived from a first set of experiments where three clones of each condition were tested, with the results represented in the graphs as mean ± standard deviation (* = p ＜ 0.05; ** = p ＜ 0.01 and *** = p ＜ 0.001). A second independent experiment yielded very similar results and is shown in S3 Fig. MFI: Mean Fluorescence Intensity.

Tethering assays have been used routinely to investigate the impact of specific proteins on the turnover and/or translation of mRNAs to which they were tethered at the 5’ or 3’ untranslated regions. These assays are dependent on the expression of fusion proteins having a small RNA binding domain, most commonly the one derived from the λ bacteriophage antiterminator protein N (λN peptide), joined to the coding sequence of the protein to be investigated. The fusion protein then is expressed in cell lines also expressing a reporter mRNA coding for a quantifiable protein product and having inserted within its untranslated regions one or more copies of the target sequence for the selected RNA binding domain, with the 19 nucleotide boxB motif being the target for the λN peptide [54,55]. Previous assays have used a tethering approach to screen for a large number of *T. brucei* proteins which are able to enhance or reduce expression of targeted mRNAs in bloodstream cells [52,53]. These assays, however, were not able to identify EIF4AI as a post-transcriptional regulator. Here we opted to develop a new tethering assay aiming to investigate the direct impact of EIF4AI on the expression of a bound reporter mRNA. This was based on a procyclic cell line, named 4213, constitutively expressing the enhanced Green Fluorescent Protein (eGFP) from a mRNA having a boxB stem loop placed within its 3’UTR (Fig 2A) and with a 5’UTR approximately 50 nucleotides long. These cells express a ~ 27 kDa fluorescent protein which is clearly seen when assayed through western blot probed with an anti-GFP antibody (S2 Fig). The GFP expression levels can also be readily quantitated through flow cytometry, as shown in Fig 2B.

To analyse the EIF4AI in the tethering assay, the cell line 4213 was next transfected with a plasmid encoding tetracycline inducible variants of the EIF4AI protein having, at their N-terminus, the λN peptide followed by the TY epitope. Transgenic cells expressing both wild-type and the DQAD mutant were generated and tested through western blots to confirm the expression of the eGFP reporter as well as the induction of the effector proteins (*λ*N-TY-EIF4AI_WT_ and *λ*N-TY-EIF4AI_DQAD_). Bands representing both the wild-type and mutant protein, with a molecular weight of ~ 50 kDa, were similarly detected using an anti-TY antibody 24 hours after tetracycline addition, with the eGFP band also identified using the anti-GFP antibody (Fig 2C). Flow cytometry was used to quantify eGFP expression in the presence of the effector proteins (+TET) and compared to non-induced samples (-TET) (Fig 2D). There was an increase in fluorescence of ~ 28% in the cell line expressing the *λ*N-TY-EIF4AI_WT_ at 48 hours after tetracycline induction, compatible with EIF4AI functioning as a post-transcriptional activator. In contrast, *λ*N-TY-EIF4AI_DQAD_ expression resulted in a decrease in GFP expression of ~ 18%, when compared to the corresponding non-induced strains (an independent experiment is shown in S3 Fig). These results confirm the expected role for EIF4AI in stimulating the expression of the eGFP reporter but indicate that this role is dependent on the integrity of the DEAD box motif and possibly its helicase/ATPase activity.

As control for the tethering assay, a cell line expressing a similar eGFP mRNA, with the same UTRs but without the boxB stem loop, was also generated (S4 Fig) and tested in the same conditions as above. The impact on the control cell line was somewhat variable between experiments, however, considering the average eGFP levels detected. Nevertheless, little or no effect on the reporter was detected after induction of the λN-TY-EIF4AI_WT_, whilst the presence of the λN-TY-EIF4AI_DQAD_ mutant either did not affect or led to a slight decrease in eGFP levels (S5 Fig). Another control used to validate the new tethering approach was performed by also investigating the effect on the reporter mRNA of the *T. brucei* EIF4E1. This eIF4E homologue has been consistently found to function as a repressor in the previously described tethering system based in *T. brucei* bloodstream forms [52,53,56]. A repressive effect was also seen here on the eGFP expression from the 4213 cell line, post EIF4E1 induction. In contrast, minor effects were observed for the reporter from the control cell line (S6 Fig).

### The RocA inhibitor impairs growth of the *T. brucei* procyclic form and impacts on the effect of the EIF4AI_WT_ expression on the levels of a reporter mRNA

**Fig 3:**
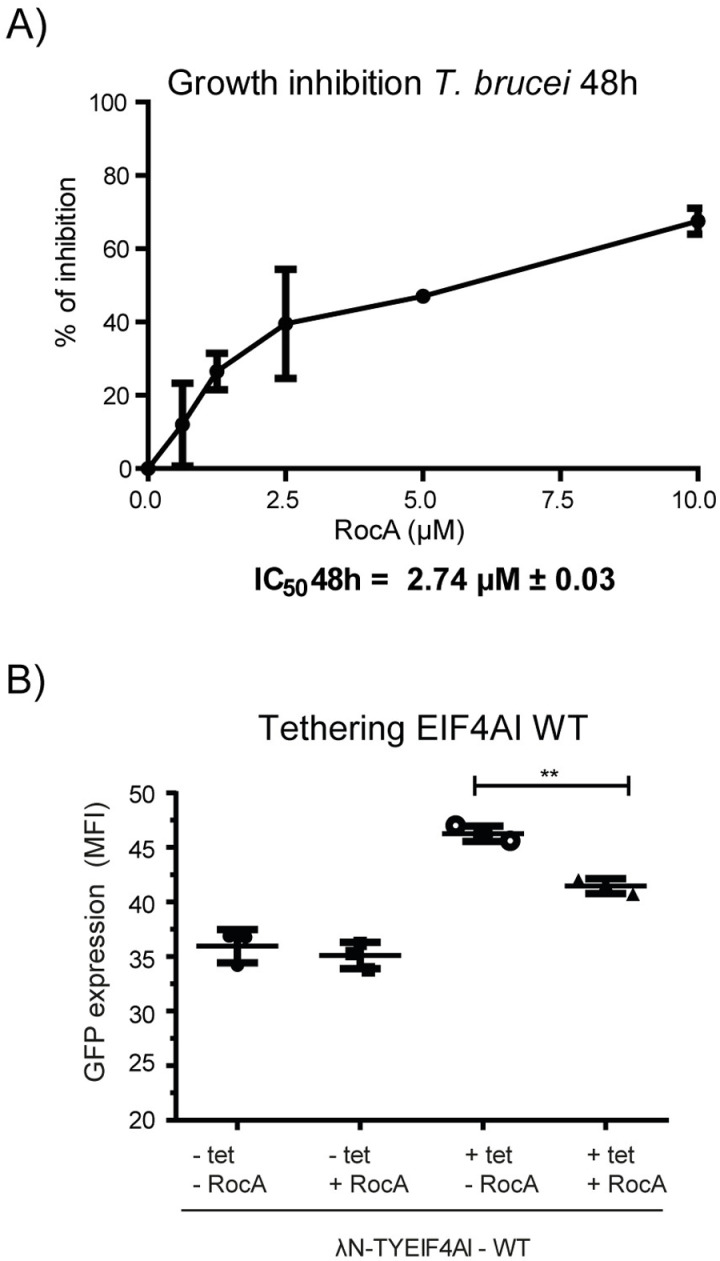
Evaluation of the RocA inhibitor on the *T. brucei* growth and EIF4AI activity. A) Growth curve assessing the effect of the RocA inhibitor on the growth of *T. brucei* procyclic cells. The results shown are representative of two independent experiments, each performed in triplicates and detailed in the S7 Fig. (B) Comparative analysis by flow cytometry of the expression of the eGFP reporter after induction of the *λ*N-TY-EIF4AI_WT_ fusion, in the presence or absence of RocA at a concentration of 1 µ M. MFI: Mean Fluorescence Intensity.

Many natural and synthetic molecules, such as hippuristanol, rocaglates, pateamine A and derivatives, have shown inhibitory effects against eIF4A and this property has been used to study its function in translation, as well as to explore their potential use as drug candidates [57]. Rocaglamide A (RocA) is a typical rocaglate that has been seen to inhibit both eIF4A as well as the DDX3 DEAD box helicase [58] and whose effect on the *Leishmania* EIF4AI has also been assessed [59]. Here, to investigate the possible use of this inhibitor to evaluate eIF4A function in the *T. brucei* 427 cell line, we first tested its effect on the growth rate of procyclic forms cultured in the presence of different concentrations of RocA. As shown in Fig 3A, representing the percentage of growth inhibition seen, an inhibitory effect was indeed observed with an IC_50_ value estimated at 2.74 µ M ± 0.03 µ M.

We then opted to assess the effect of RocA on the reporter eGFP expression in the tethering assay with the *λ*N-TY-EIF4AI_WT_ protein (Fig 3B). The inhibitor, at 1 µ M, had no effect on the levels of the GFP reporter on cells which were not expressing the fusion protein (-TET), however it did lead to a reduction in GFP levels when *λ*N-TY-EIF4AI_WT_ was induced (+TET/ + RocA), with an overall ~10% decrease in the eGFP expression when compared to control (+TET/-RocA). These results confirm a requirement for the EIF4AI activity in the tethering assays but, nevertheless, some stimulation by the *λ*N-TY-EIF4AI_WT_ on the reporter expression remained even in the presence of RocA.

### Reduced *T. brucei* susceptibility to RocA explained by differences in its eIF4A binding pocket

**Fig 4:**
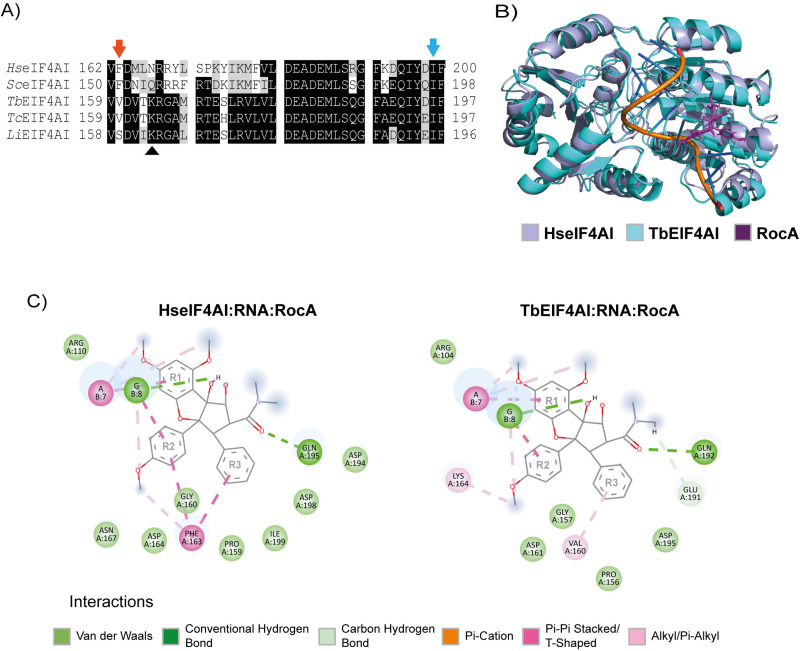
Comparative evaluation of the interaction between the human and *T. brucei* eIF4A homologues with RNA and RocA. (A) Alignment comparing the RocA binding region from selected eIF4AI sequences: *Homo sapiens* (Hs), *Saccharomyces cerevisiae* (Sc), *Trypanosoma brucei* (Tb), *Trypanosoma cruzi* (Tc) and *Leishmania infantum* (Li). The arrows indicate the relevant phenylalanine (F163 in the human eIF4AI; red arrow) and isoleucine (I199 in humans; blue arrow) implicated in the interaction with RocA, whilst the *Leishmania* K163 residue is indicated with the symbol ▲. The complete sequence alignment can be found in the S8 Fig. (B) Overlay of the *Tb*EIF4AI structural model with the solved model for *Hs*eIF4AI (PDB: 5ZC9). (C) 2D maps of the RocA binding pocket, based on the structure of the human eIF4AI:RNA interacting with RocA as well as the model for the TbEIF4AI:RNA:RocA complex*.*

The RocA IC_50_ estimated for *T. brucei* indicates a much reduced susceptibility to this inhibitor, when compared with mammalian HEK293 cells, with an IC_50_ calculated at 3.68 ± 0.51 nM [60]. This difference is noteworthy, especially considering potential studies investigating the use of related chemicals to inhibit eIF4A function in these parasites. We thus decided to investigate whether differences in the corresponding eIF4A sequences/structures could possibly lead to the variations observed regarding the RocA inhibitory effect. Two residues, phenylalanine 163 (F163) and isoleucine 199 (I199), in human eIF4AI numbering, have been reported as important for resistance to RocA since the presence of two mutations in those residues in *Aglaia* (F163L and I199M) avoid self-toxicity. The F163 residue, in particular, is considered a determinant for sensitivity to rocaglates, including Rocaglamide A, and substitutions can directly interfere with the way the inhibitor interacts with the binding pocket, affecting the eIF4A susceptibility to the drug [60]. To investigate the conservation of both residues in trypanosomatids we opted here to first compare the equivalent sequences from three different species: *T. brucei*, *T. cruzi* and *Leishmania infantum*. The I199A residue is conserved in the EIF4AI sequences from the three trypanosomatid species (Fig 4A and S8 Fig), but F163 diverges in all. In both *T. brucei* and *T. cruzi* species it is replaced by a valine (V160).

In order to provide insights into how EIF4AI establishes its interactions with the inhibitor, we opted to model the interactions between the *T. brucei* EIF4AI with RNA and RocA based on the solved structure of the human eIF4AI:RNA:RocA. A AlphaFold2 structural model was then generated for the *T. brucei* EIF4AI and this model was overlayed with the human structure (Fig 4B). The model was found with a greater than 95% residue distribution in allowed regions of the Ramachandran plot, classifying it as an excellent model, with Root Mean Square Deviation (RMSD) values indicating significant structural similarity between the modelled protein and the human eIF4AI (S9 Fig). We then analysed, in a structural context, the residues that make up the RocA binding pocket within the *T. brucei* EIF4AI and found that the absence of phenylalanine in this pocket plus the valine (V160) substitution generates a hydrophobic (alkyl) interaction between this valine residue and one of the RocA rings (R3-ring) (Fig 4C and S10 Fig). It is a weaker interaction when compared to the one formed between RocA and F163 in the eIF4A binding pocket found in humans and other eukaryotes. F163 forms intermolecular interactions of the Pi-T-shaped and Pi-Pi-stacked types with two RocA rings (R2 and R3), which stabilizes the ligand in the binding pocket. The weaker interaction is compatible with the reduced *T. brucei* susceptibility observed for this inhibitor.

### Differences in amino acids involved in RocA binding to eIF4A are associated with different inhibitory responses seen for *T. brucei* and *L. infantum*

**Fig 5:**
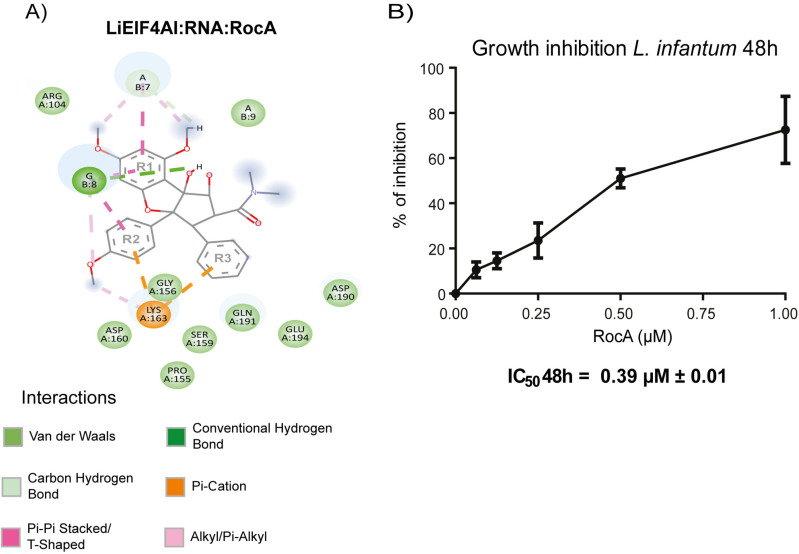
Evaluation of the RocA interaction with the *L. infantum* EIF4AI and its impact on the parasite growth in culture. (A) 2D map of the RocA binding pocket on the *L. infantum* EIF4AI, based on the model for the LiEIF4AI:RNA:RocA complex built as described in Fig 4 for the *T. brucei* EIF4AI. (B) Growth curve assessing the effect of the RocA inhibitor on the growth of *L. infantum* promastigote cells. The results shown are representative of three independent experiments, each performed in triplicates and detailed in the S7 Fig*.*

The alignment comparing the human, *Trypanosoma* and *Leishmania* eIF4A sequences reveals a different substitution for the F163 from the human eIF4AI in the *L. infantum* protein, with a serine found in the corresponding position (S159). This implies a shorter and more hydrophilic side chain for the *L. infantum* EIF4AI, when compared with the one provided by the V160 residue found at the same position in its *T. brucei* orthologue. Considering then the potential for a different interaction with RocA for the *L. infantum* EIF4AI, and changes in the parasite’s susceptibility to the inhibitor, we decided to also model the *L. infantum* EIF4AI:RNA:RocA interactions following the same procedure described for the *T. brucei* EIF4AI, with equivalent quality parameters found for the *Leishmania* protein (S9 Fig). Indeed, a different profile is observed regarding the RocA interaction with the *L. infantum* EIF4AI (Fig 5A and S10 Fig). In this case, the presence of the S159 residue in the *L. infantum* EIF4AI, despite contributing to weaker interactions with RocA, appears to be compensated by it still allowing another residue, lysine 163 (K163), access to the RocA ligand. In the *L. infantum* EIF4AI, the K163 residue can thus also interact with two RocA rings (R2 and R3), through interactions of the pi-cation type, leading to a more stable and overall stronger interaction with the ligand, when compared with the *T. brucei* EIF4AI*.*

Based on the *in silico* analyses, we then opted to evaluate the effect of RocA on the *L. infantum* growth*,* using its promastigote developmental form (Fig 5B). Indeed, this inhibitor was found to impair the parasite growth at lower concentrations than observed for *T. brucei*. At 0.39 ± 0.01 µ M, the RocA IC_50_ estimated for *L. infantum* is four to five fold lower than the one calculated for *T. brucei*, despite still being much higher than the one defined for mammalian cells. This difference in the RocA IC_50_ between the two trypanosomatid species investigated here is noteworthy and might be explained by the differences in the availability of amino acid residues in the EIF4AI binding pocket seen for these species. Nevertheless, further experimental validation of the role of the residues discussed here on the RocA binding is still needed.

## Discussion

The association of the trypanosomatid EIF4AI with the eIF4F-like complexes based on the EIF4E4/EIF4G3 and EIF4E3/EIF4G4 subunits has been shown by co-precipitation studies from whole cytoplasmic extracts performed both in *T. brucei* and *Leishmania* [31,33,45,61,62], as well as by *in vitro* pull-down assays confirming the direct interaction of the *Leishmania* EIF4AI with both EIF4G3 and EIF4G4 [31,63]. The *in vitro* assays, however, did indicate differences in the interaction between EIF4AI and the two eIF4G subunits [31], despite both EIF4G3 and EIF4G4 having conserved motifs at sequences shown in the yeast eIF4G to mediate the binding to its eIF4A partner [27], and the recent data from *T. brucei* also suggests a greater association *in vivo* between EIF4AI and the EIF4E4/EIF4G3 based complex [33]. Here our results, the first using the tagged EIF4AI as bait, confirm its strong association with the EIF4E4/EIF4G3 based complex, whilst also indicating an ability to associate with the complex formed by the EIF4E3/EIF4G4 subunits. The data, however, does not support any association between EIF4AI and the other eIF4F-like complexes from trypanosomatids, despite the EIF4AI co-precipitation previously observed with two other eIF4G homologues, EIF4G1 and EIF4G2 [64].

The helicase activity of eIF4A is essential for protein synthesis and cell viability, and mutants that lack this activity can induce a dominant negative effect. From previous studies, mutations targeting the motif II (DEAD) interfere with the EIF4AI ATPase and helicase activities without affecting RNA binding (reviewed in [22]. These mutations are not expected to impact directly on the EIF4AI interactions, although they can lead to structural rearrangements which would interfere with them. Indeed, the results reported here do show an important impact for the DQAD mutation upon the EIF4AI interactions, since the same mutation leads to contrasting effects in regard to the association between EIF4AI and the EIF4E4/EIF4G3 and EIF4E3/EIF4G4 complexes. Whilst the DQAD mutant is clearly impaired in its association with the EIF4E4/EIF4G3 pair, the mutation might also stabilize an otherwise more transient interaction with the complex based on the EIF4E3/EIF4G4 subunits and other translation factors. These differences likely reflect distinct functional properties associated with the eIF4A activity within these complexes.

The results with the mutant EIF4AI imply that the EIF4E4/EIF4G3 complex most likely interacts with EIF4AI when it is bound to ATP. Based on structural data (reviewed in [8]), this would require a closed conformation for EIF4AI. A more stable interaction would also be compatible with both EIF4E4/EIF4G3 subunits being more efficiently co-precipitated with the native EIF4AI. The EIF4E4/EIF4G3 complex has been shown to preferentially associate with mRNAs having very short 5’UTRs [32,33] which presumably require less RNA helicase activity and little or no scanning by the small ribosomal subunit. eIF4A homologues in yeast and humans, and in the more diverged *Giardia* parasite, have been shown to directly interact with subunits of the eIF3 complex [37,39,40,42], implying functions not previously defined. In mammals, at least, the direct eIF3 interactions have been found to involve a second eIF4A molecule, localized opposite to eIF4F on the small ribosomal subunit, and which might be required for the efficient mRNA scanning [42]. A speculative proposal for EIF4AI based on the results shown here, within the EIF4E4/EIF4G3 complex, would be for it to be preferentially supporting the adequate ribosomal placement along the mRNA, rather than helping mRNA scanning. A requirement for ATP at this stage is supported by recent work in yeast which confirmed the requirement for eIF4A, and ATP hydrolysis, for the efficient ribosome recruitment to the capped mRNAs [38].

The tethering assay suggests that the EIF4AI_DQAD_ mutant might be non-functional, consistent with a lack of interaction with EIF4E4/EIF4G3, but this is not compatible with a dominant negative phenotype. In contrast, the co-precipitation of the mutant protein with a second eIF4F-like complex, EIF4E3/EIF4G4, might indicate a stabilization by the DQAD mutation of the interaction between EIF4AI and EIF4E3/EIF4G4, which otherwise is not seen. Since other translation-related factors and ribosomal proteins are also found with the mutant protein, it is possible that the binding of the mutant protein to the EIF4E3/EIF4G4 complex might prevent its activity when already bound to the mRNA and to other translation related proteins. This is more compatible with the dominant mutant phenotype previously observed for the DQAD mutation in *T. brucei* [29]. It also is in agreement with the multiple cycles of EIF4AI binding and release to the RNA and/or eIF4F, which are expected to be required for the helicase activity to proceed [8,10]. The mutant protein, by remaining bound to the EIF4E3/EIF4G4 complex, is likely to prevent it to function effectively and should also compete with the endogenous non-mutated EIF4AI, which otherwise would still be capable of providing eIF4F-like functionality.

The specific association between the mutated EIF4AI and the EIF4E3/EIF4G4 complex is reinforced by its co-precipitation with RNA helicases (HEL67, DED1, DBP2B) as well as other proteins, such as DRBD2 and PABP2, previously found to be preferentially enriched with EIF4E3/EIF4G4 [33]. Both HEL67 and the related DED1 are homologues of the yeast ded1p and human DDX3, whose orthologues in *Leishmania* have been previously studied [65]. The Ded1/DDX3 helicases are enzymes universally conserved among eukaryotes [12] and have been found to be preferentially required during the ribosome scanning of mRNAs having more structured 5’ UTRs [10,13]. The HEL67/DED1 association with the EIF4E3/EIF4G4 pair might then suggest a role during the unwinding activity of the longer 5’UTRs seen preferentially found in the mRNAs co-precipitated with this complex [33], a role which nevertheless might still require EIF4AI. As to the reasons why the EIF4E3/EIF4G4 complex is not found with the native EIF4AI, a possible explanation would be the transient nature of this association. The large differences in abundance between EIF4AI and its binding partner EIF4G4, based on previous quantitations [29,31], also implies that only a minor fraction of EIF4AI would be bound to an eIF4G partner, further impacting on the proper detection of this loosely bound partner. Similar reasons might also explain the lack of other eIF4G homologues co-precipitating with EIF4AI, although their lack of co-precipitation with the DQAD mutant would also imply relevant differences in comparison with EIF4G4.

A number of recent studies have investigated translation inhibitors targeting eIF4A, focusing on both the validation of novel inhibitors as well as on the investigation of mechanisms associated with eIF4A inhibition and function [57,60,66–70]. RocA and other rocaglates are known to interfere with eIF4A activity by increasing its affinity and clamping to the RNA substrate, leading to a sequestration of the eIF4A and eIF4F available and to the inhibition of translation [67,69]. The RocA effect on the tethering assay seen here does not show a major impact by the inhibitor on the stimulation of the reporter expression induced by EIF4AI. Although this could be a consequence of the reduced sensitivity to the drug by the *T. brucei* EIF4AI, it might also reflect the increased EIF4AI levels, induced by the ectopic gene, or the fact that RocA might not significantly impact the EIF4AI function when bound to EIF4E4/EIF4G3, the complex most likely found to be associated with the EIF4AI activity in the tethering assay. Several recent papers have also investigated the potential use of specific inhibitors to target eIF4A from different pathogens [59,71,72]. Silvestrol, another roclagate, has been specifically tested with both *T. brucei* and *Leishmania amazonensis* [72], with *T. brucei* considered a susceptible species, due to a much greater susceptibility to silvestrol than the susceptibility observed here for RocA, whilst *L. amazonensis* was considered resistant to the drug. However, silvestrol is a larger molecule and has an additional 1,4-dioxane moiety added to the basic rocaglate skeleton that is missing from RocA. This has been suggested to mediate additional interactions between silvestrol and eIF4A beyond the RNA binding pocket [72] and might explain a greater interaction for silvestrol, and greater inhibitory potential, than RocA for *T. brucei* cells, at least. The differences in the results for the *Leishmania* cells are harder to explain, but the previous work investigated the susceptibility to silvestrol in intracellular amastigotes and used lower concentration of the drugs. In contrast, our assays tested extracellular promastigotes with higher RocA concentrations, so one possibility would be for differences in intracytoplasmic drug availability within the *Leishmania* cells lead to the distinct results observed. An early evaluation of RocA did find a higher EC value for RocA (16.5 μM) with *L. infantum* promastigotes [73], but the reason for the observed discrepancy is not currently understood. Overall, these results and the others reported here help clarify important aspects regarding EIF4AI function during translation in trypanosomatids, with relevant insights revealed regarding the dynamics of mRNA translation in these and other eukaryotes and with implications for drug discovery research aiming to identify new targets for antiparasitic drugs.

## Supporting information

S1 Table Proteins co-precipitated with the EIF4AI wild-type. Only proteins represented by a minimum of two peptides in at least two of the replicates from the bait and with an enrichment ratio in comparison with the control equal to or greater than 1.5 were considered and included in the table.
(XLSX)

S2 Table Proteins co-precipitated with the EIF4AI DQAD mutant. Only proteins represented by a minimum of two peptides in at least two of the replicates from the bait and with an enrichment ratio in comparison with the control equal to or greater than 1.5 were considered and included in the table.
(XLSX)

S3 Table Volcano plot data for the EIF4AI-DQAD/EIF4AI-WT comparative analysis. (XLSX)

S1 Raw Images Raw images with full sized results from the western-blots used to generate Fig 2C and the supporting figures S1 Fig, S2 Fig, S4 Fig, S5 Fig and S6 Fig. (PDF)

S1 Fig Expression and immunoprecipitation of the HA-tagged EIF4AI proteins. The left panels show western blots confirming the expression in transgenic lines of the EIF4AI_WT_-HA and EIF4AI_DQAD_-HA proteins, each tagged with six copies of the HA epitope, with the same blot probed with an anti-HA monoclonal antibody and with polyclonal anti-serum against the native EIF4AI. The western blot on the right panel assesses the immunodetection with anti-HA of various samples from the immunoprecipitation experiments using anti-HA magnetic beads. These include the cytoplasmic fractions or lysates (L) of cell lines expressing *T. brucei* EIF4AI, in its wild-type (WT) and mutant form (DQAD), as well as the immunoprecipitated (IP) samples and the non-bound or flow through (FT) fractions, with the parental cell line (29–13) used as negative control.
(PDF)

S2 Fig Schematic representation of the tethering construct and evaluation of the eGFP expression in transfected *T. brucei* cells. The figure shows a schematic representation of the plasmid segment encompassing the reporter construct as well as the corresponding mRNA. This encodes the eGFP reporter plus boxB motif, represented by a stem loop placed within the mRNA 3’UTR. Binding of the λN-TY-EIF4AI protein to the boxB motif is also represented. The constitutive expression of eGFP was detected by western blot (panels below) and compared to the non-transfected parental cell line, with the detection of the chaperone BiP used as loading control.
(PDF)

S3 Fig Independent tethering assay to evaluate the EIF4AI impact on the expression of a reporter mRNA. Quantitative analysis of eGFP expression 48 hours after tetracycline induction for the second set of experiments carried out as described for Fig 2E.
(PDF)

S4 Fig Plasmid construct, mRNA and eGFP expression analysis for the control cell line used in the tethering assays. Schematic representations of the plasmid segment encompassing the control reporter construct (p4212) as well as the corresponding mRNA, encoding the eGFP reporter without any boxB motif within the mRNA 3’UTR, are shown on the left. The constitutive expression of eGFP in the corresponding transgenic cell line was confirmed through the western blots shown, assessing also the non-transfected cell line, with the detection of the chaperone BiP used as loading control. The histograms on the left represent the eGFP expression detected by flow cytometry in procyclic cells from non-transfected *T. brucei* and the transgenic 4212 cell line.
(PDF)

S5 Fig Evaluation of the effect of the tethered EIF4AI, wild type and DQAD mutant, on the eGFP reporter from a control cell line. Results from two different experiments are shown, with the first experiment, shown on top, evaluated through western blot probed with the anti-TY and anti-GFP monoclonal antibodies. It confirms the expression of both the *λ*N-TY- EIF4AI_WT_ and *λ*N-TY-EIF4AI_DQAD_ in representative transgenic clones induced (+tet) or not induced (-tet) with tetracycline for 24 hours, and the effect on the eGFP expression. The same experiment was also evaluated through flow-cytometry, for a quantitative analysis of eGFP expression 48 hours after tetracycline induction. Quantitative results only for a second independent experiment are shown on the bottom. Three clones of each condition were tested for each experiment, with the results represented in the graphs as mean ± standard deviation (* = p ＜ 0.05). MFI: Mean Fluorescent Intensity.
(PDF)

S6 Fig Evaluation of the tethered EIF4E1, used as a control, on the expression of the eGFP reporter mRNA. The effect of the *λ*N-TY-EIF4E1 expression on the eGFP reporter encoded by the mRNA with the boxB motif on its 3’UTR (from the 4213 cell line) is shown on the left. The western blots on top, probed with the anti-TY monoclonal antibody, confirm the expression of *λ*N-TY- EIF4E1 in representative transgenic clones induced (+tet, 24 and 48h) or not induced (0h) with tetracycline, with the BiP chaperone used as loading control. The quantitative analyses of the eGFP expression 24 and 48 hours after tetracycline induction, for two independent experiments, are shown below. Equivalent experiments, shown on the right, were performed in order to assess the effect of the *λ*N-TY-EIF4E1 expression on the eGFP reporter encoded by the control mRNA (from the 4212cell line). Three clones were tested for each experiment from each condition, with the results represented mean ± standard deviation.
(PDF)

S7 Fig Rocaglamide (RocA) effect on the growth of both *Trypanosoma brucei* and *Leishmania infantum.* For all the experiments, each spot represents an average of cell-density values from three different growth curves set up in parallel. These values were measured 48 hours after the cells were incubated with the RocA inhibitor and were used to define the growth inhibition results shown in Figures 3A and 5B. The experiments with the *T, brucei* procyclic cells are shown on the left, while on the right are shown those performed with the *L. infantum* promastigotes.
(PDF)

S8 Fig Multiple sequence alignment comparing the human and yeast eIF4AI sequences with the *Leishmania* and *Trypanosoma* EIF4AI orthologues. The alignment was performed using the MAFFT software, with amino acids identical in 60% or more of the sequences highlighted in black, whilst amino acids defined as similar in 60% or more of the sequences shown in grey.
(PDF)

S9 Fig Assessment of the quality of the models generated for the interactions between the *T. brucei* and *Leishmania* EIF4AI with RNA and RocA. Ramachandran plot scores for the modelled EIF4AI orthologues from *T. brucei* and *Leishmania* are shown on top. Values for all residues in allowed regions are derived from the sum of the residues in most favoured regions with those in additionally allowed and generously allowed regions. The Root Mean Square Deviation (RMSD) analysis is shown below. The RMSD is 0 for identical structures and increases with greater differences between the two structures. A RMSD near zero in the alignment between structures indicates significant structural similarity.
(PDF)

S10 Fig 3D maps of the RocA binding pocket, based on the structure of the human eIF4AI:RNA interacting with RocA as well as the model for the TbEIF4AI:RNA:RocA and LiEIF4AI:RNA:RocA complexes. Individual maps are represented with the colour codes indicated in the figure.
(PDF)
