# Supplementary material for: Distinct modes of interaction within eIF4F-like complexes and susceptibility to the RocA inhibitor for the Trypanosoma brucei EIF4AI translation initiation factor
Source: PLoS One. 2025 May 9;20(5):e0322812. doi: 10.1371/journal.pone.0322812 (PMC12063893; doi:10.1371/journal.pone.0322812)
Supplement: S1 Raw Images — (PDF) [file pone.0322812.s004.pdf]

Fig 2C - RAW DATA

T. brucei (4213 cell line)

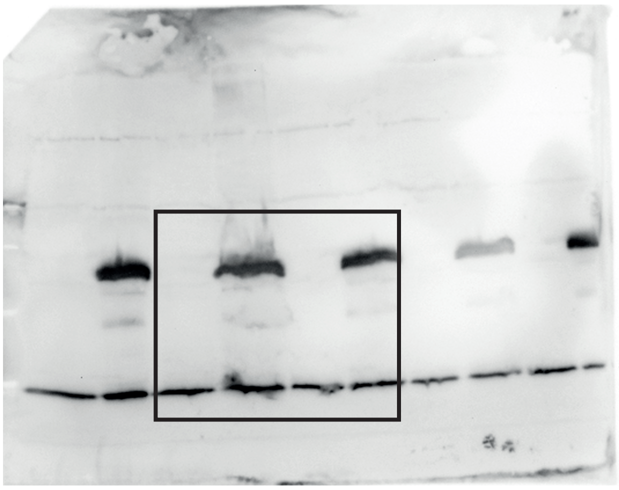

S1 Fig - RAW DATA

T. brucei (29-13 cell line)

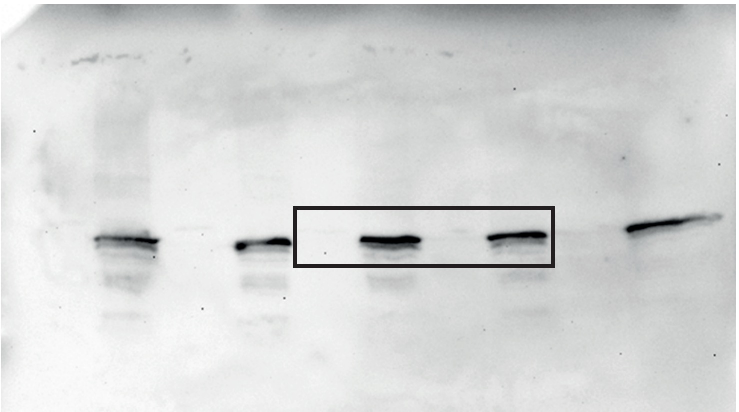

anti-HA

T. brucei (29-13 cell line)

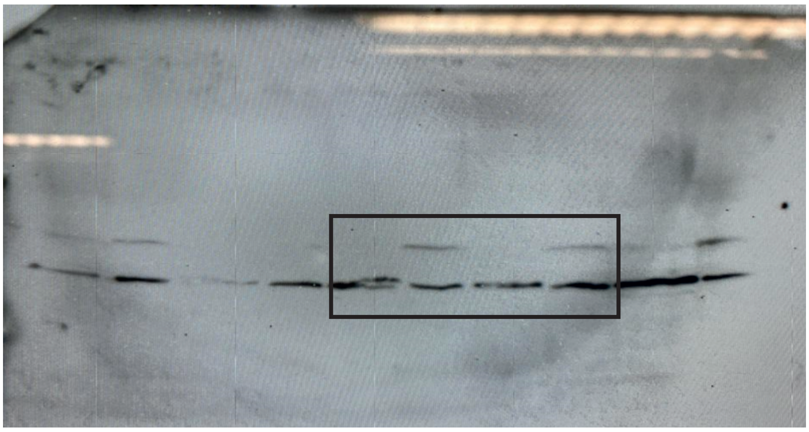

anti-EIF4A

Immunoprecipitations

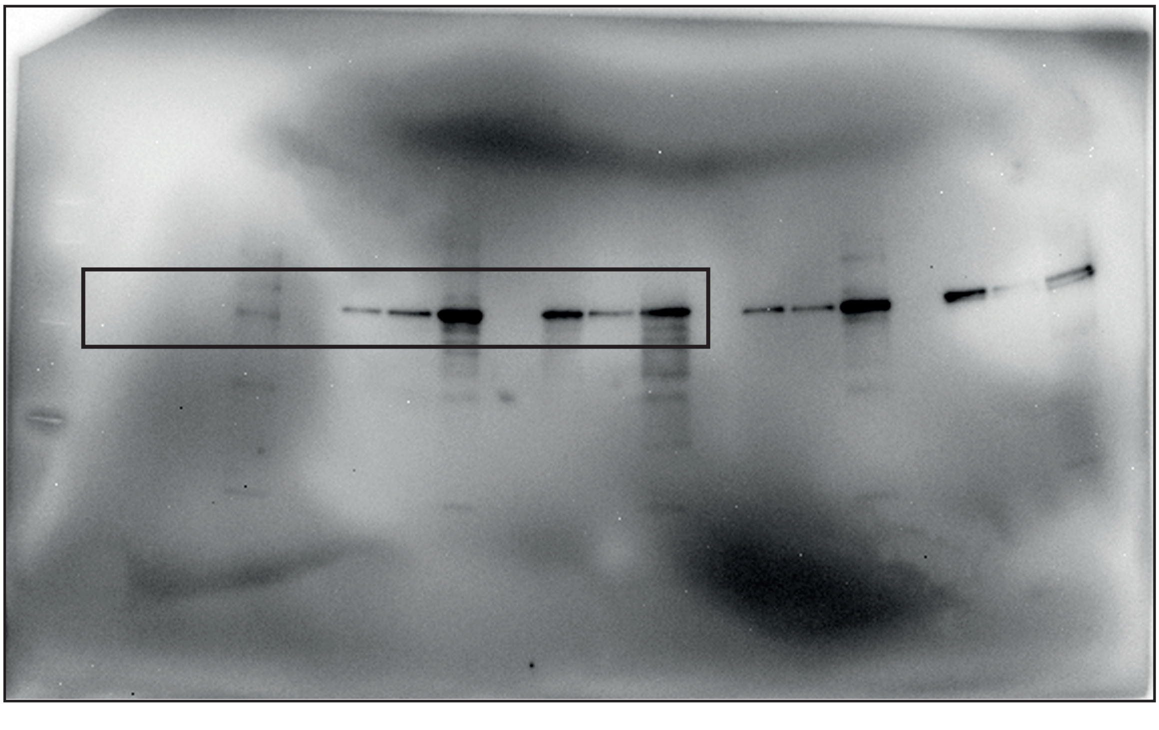

anti-HA

S2 Fig - RAW DATA

T. brucei (4213 cell line)

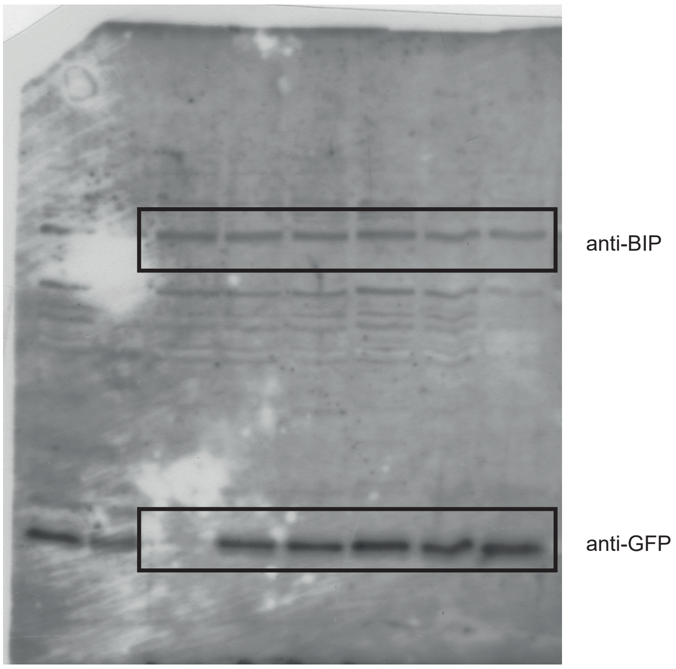

S4 Fig - RAW DATA

T. brucei (4213 cell line)

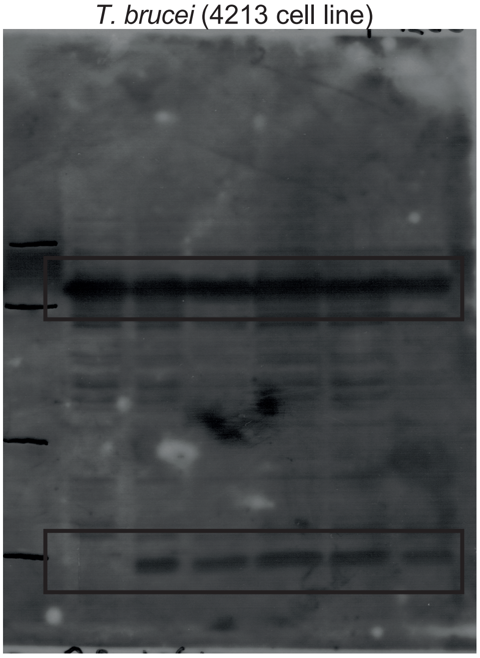

S5 Fig - RAW DATA

T. brucei (4212 cell line)

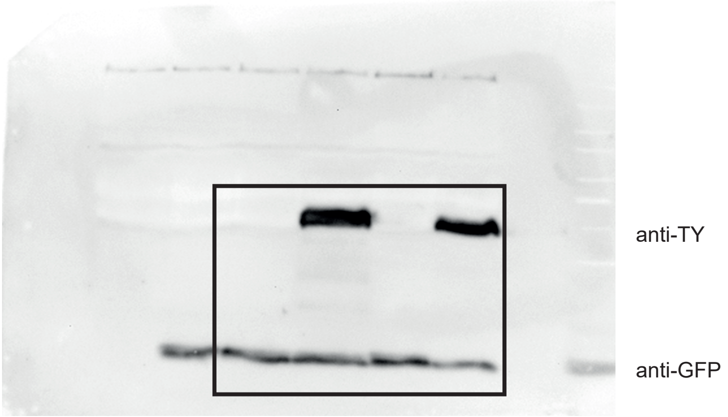

S6 Fig - RAW DATA

T. brucei (4213 cell line)

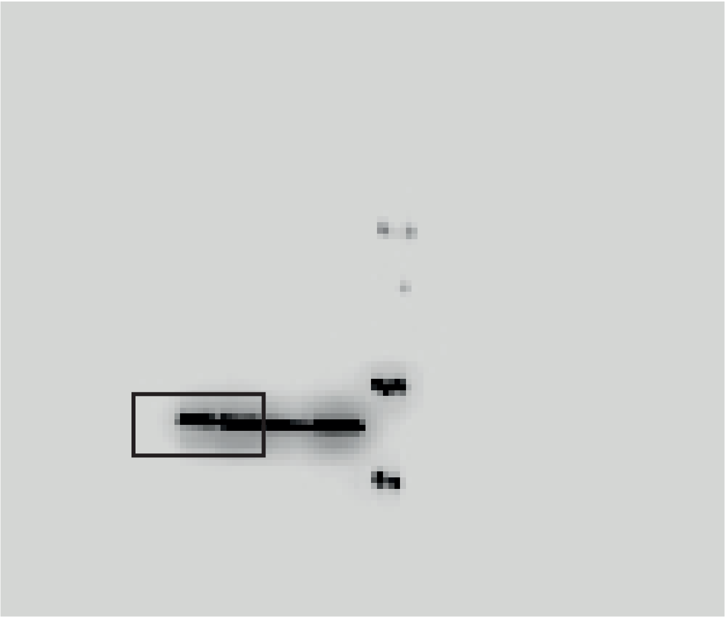

anti-TY

T. brucei (4212 cell line)

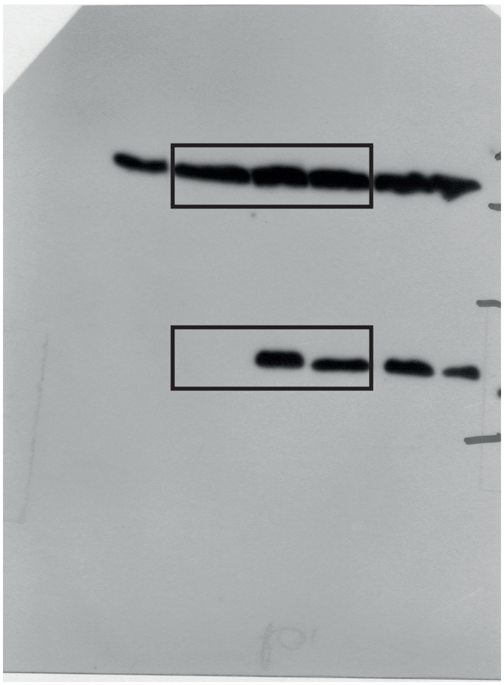

anti-BiP

anti-TY
