# Supplementary material for: Distinct modes of interaction within eIF4F-like complexes and susceptibility to the RocA inhibitor for the Trypanosoma brucei EIF4AI translation initiation factor
Source: PLoS One. 2025 May 9;20(5):e0322812. doi: 10.1371/journal.pone.0322812 (PMC12063893; doi:10.1371/journal.pone.0322812)
Supplement: S1 Fig — The left panels show western blots confirming the expression in transgenic lines of the EIF4AIWT-HA and EIF4AIDQAD-HA proteins, each tagged with six copies of the HA epitope, with the same blot probed with an anti-HA monoclonal antibody and with polyclonal anti-serum against the native EIF4AI. The western blot on the right panel assesses the immunodetection with anti-HA of various samples from the immunoprecipitation experiments using anti-HA magnetic beads. These include the cytoplasmic fractions or lysates (L) of cell lines expressing T. brucei EIF4AI, in its wild-type (WT) and mutant form (DQAD), as well as the immunoprecipitated (IP) samples and the non-bound or flow through (FT) fractions, with the parental cell line (29–13) used as negative control. (PDF) [file pone.0322812.s005.pdf]

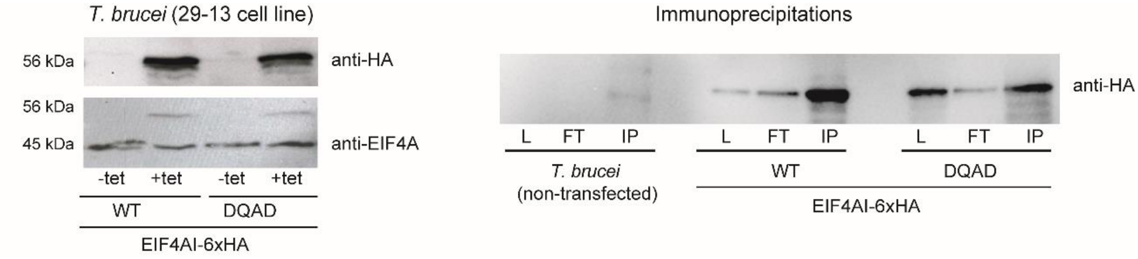

S1 Fig – Expression and immunoprecipitation of the HA-tagged EIF4AI proteins. The left panels show western blots confirming the expression in transgenic lines of the EIF4AI$_{WT}$-HA and EIF4AI$_{DQAD}$-HA proteins, each tagged with six copies of the HA epitope, with the same blot probed with an anti-HA monoclonal antibody and with polyclonal anti-serum against the native EIF4AI. The western blot on the right panel assesses the immunodetection with anti-HA of various samples from the immunoprecipitation experiments using anti-HA magnetic beads. These include the cytoplasmic fractions or lysates (L) of cell lines expressing *T. brucei* EIF4AI, in its wild-type (WT) and mutant form (DQAD), as well as the immunoprecipitated (IP) samples and the non-bound or flow through (FT) fractions, with the parental cell line (29-13) used as negative control.
