# Supplementary material for: Distinct modes of interaction within eIF4F-like complexes and susceptibility to the RocA inhibitor for the Trypanosoma brucei EIF4AI translation initiation factor
Source: PLoS One. 2025 May 9;20(5):e0322812. doi: 10.1371/journal.pone.0322812 (PMC12063893; doi:10.1371/journal.pone.0322812)
Supplement: S2 Fig — The figure shows a schematic representation of the plasmid segment encompassing the reporter construct as well as the corresponding mRNA. This encodes the eGFP reporter plus boxB motif, represented by a stem loop placed within the mRNA 3’UTR. Binding of the λN-TY-EIF4AI protein to the boxB motif is also represented. The constitutive expression of eGFP was detected by western blot (panels below) and compared to the non-transfected parental cell line, with the detection of the chaperone BiP used as loading control. (PDF) [file pone.0322812.s006.pdf]

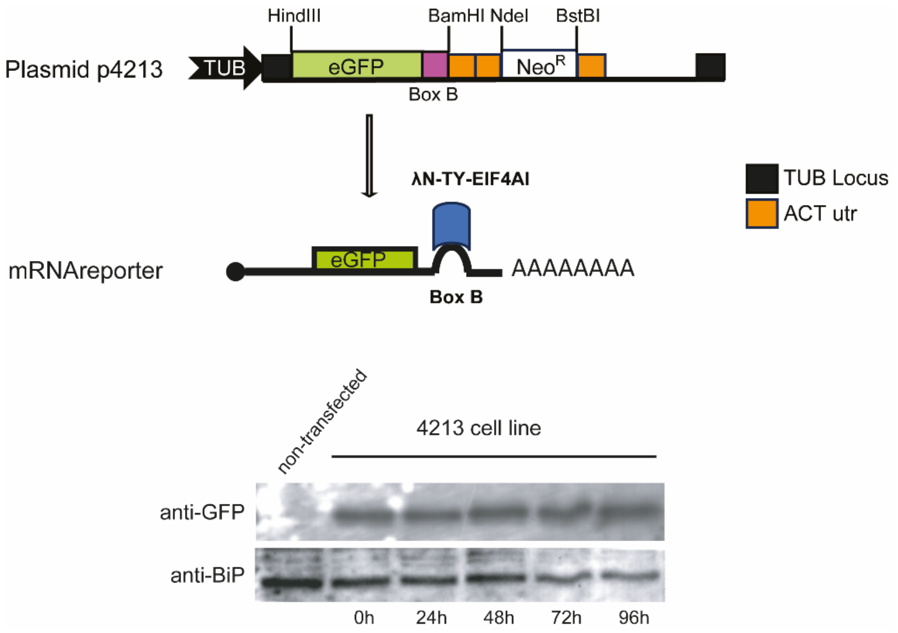

S2 Fig – Schematic representation of the tethering construct and evaluation of the eGFP expression in transfected *T. brucei* cells. The figure shows a schematic representation of the plasmid segment encompassing the reporter construct as well as the corresponding mRNA. This encodes the eGFP reporter plus boxB motif, represented by a stem loop placed within the mRNA 3'UTR. Binding of the λN-TY-EIF4AI protein to the boxB motif is also represented. The constitutive expression of eGFP was detected by western blot (panels below) and compared to the non-transfected parental cell line, with the detection of the chaperone BiP used as loading control.
