# Supplementary material for: Distinct modes of interaction within eIF4F-like complexes and susceptibility to the RocA inhibitor for the Trypanosoma brucei EIF4AI translation initiation factor
Source: PLoS One. 2025 May 9;20(5):e0322812. doi: 10.1371/journal.pone.0322812 (PMC12063893; doi:10.1371/journal.pone.0322812)
Supplement: S3 Fig — Quantitative analysis of eGFP expression 48 hours after tetracycline induction for the second set of experiments carried out as described for Fig 2E. (PDF) [file pone.0322812.s007.pdf]

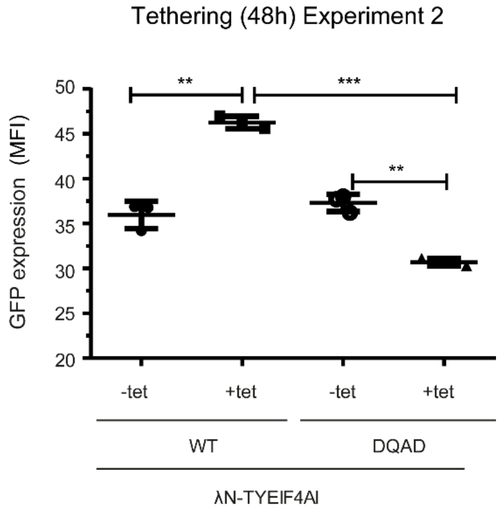

S3 Fig – Independent tethering assay to evaluate the EIF4AI impact on the expression of a reporter mRNA. Quantitative analysis of eGFP expression 48 hours after tetracycline induction for the second set of experiments carried out as described for Figure 2E.
