# Supplementary material for: Distinct modes of interaction within eIF4F-like complexes and susceptibility to the RocA inhibitor for the Trypanosoma brucei EIF4AI translation initiation factor
Source: PLoS One. 2025 May 9;20(5):e0322812. doi: 10.1371/journal.pone.0322812 (PMC12063893; doi:10.1371/journal.pone.0322812)
Supplement: S4 Fig — Schematic representations of the plasmid segment encompassing the control reporter construct (p4212) as well as the corresponding mRNA, encoding the eGFP reporter without any boxB motif within the mRNA 3’UTR, are shown on the left. The constitutive expression of eGFP in the corresponding transgenic cell line was confirmed through the western blots shown, assessing also the non-transfected cell line, with the detection of the chaperone BiP used as loading control. The histograms on the left represent the eGFP expression detected by flow cytometry in procyclic cells from non-transfected T. brucei and the transgenic 4212 cell line. (PDF) [file pone.0322812.s008.pdf]

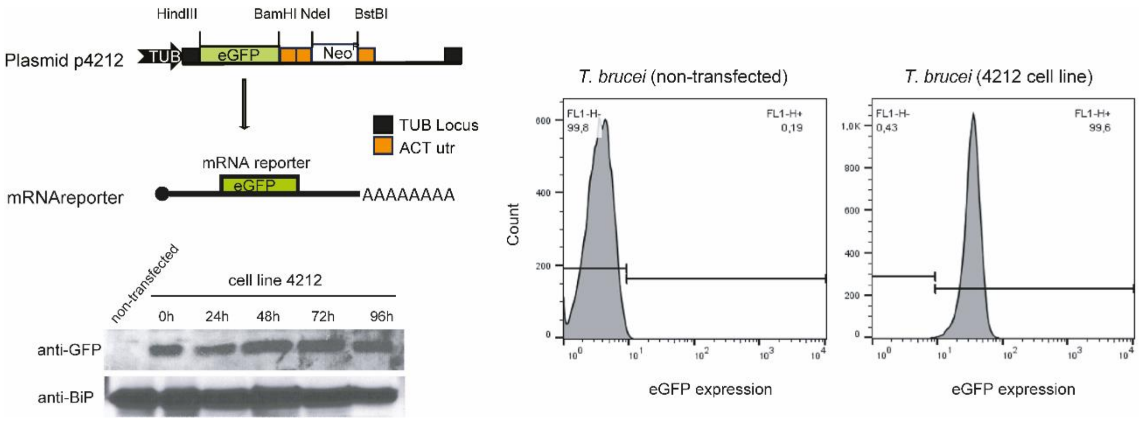

S4 Fig – Plasmid construct, mRNA and eGFP expression analysis for the control cell line used in the tethering assays. Schematic representations of the plasmid segment encompassing the control reporter construct (p4212) as well as the corresponding mRNA, encoding the eGFP reporter without any boxB motif within the mRNA 3'UTR, are shown on the left. The constitutive expression of eGFP in the corresponding transgenic cell line was confirmed through the western blots shown, assessing also the non-transfected cell line, with the detection of the chaperone BiP used as loading control. The histograms on the left represent the eGFP expression detected by flow cytometry in procyclic cells from non-transfected *T. brucei* and the transgenic 4212 cell line.
