# Supplementary material for: Distinct modes of interaction within eIF4F-like complexes and susceptibility to the RocA inhibitor for the Trypanosoma brucei EIF4AI translation initiation factor
Source: PLoS One. 2025 May 9;20(5):e0322812. doi: 10.1371/journal.pone.0322812 (PMC12063893; doi:10.1371/journal.pone.0322812)
Supplement: S5 Fig — Results from two different experiments are shown, with the first experiment, shown on top, evaluated through western blot probed with the anti-TY and anti-GFP monoclonal antibodies. It confirms the expression of both the λN-TY- EIF4AIWT and λN-TY-EIF4AIDQAD in representative transgenic clones induced (+tet) or not induced (-tet) with tetracycline for 24 hours, and the effect on the eGFP expression. The same experiment was also evaluated through flow-cytometry, for a quantitative analysis of eGFP expression 48 hours after tetracycline induction. Quantitative results only for a second independent experiment are shown on the bottom. Three clones of each condition were tested for each experiment, with the results represented in the graphs as mean ± standard deviation (* = p ＜ 0.05). MFI: Mean Fluorescent Intensity. (PDF) [file pone.0322812.s009.pdf]

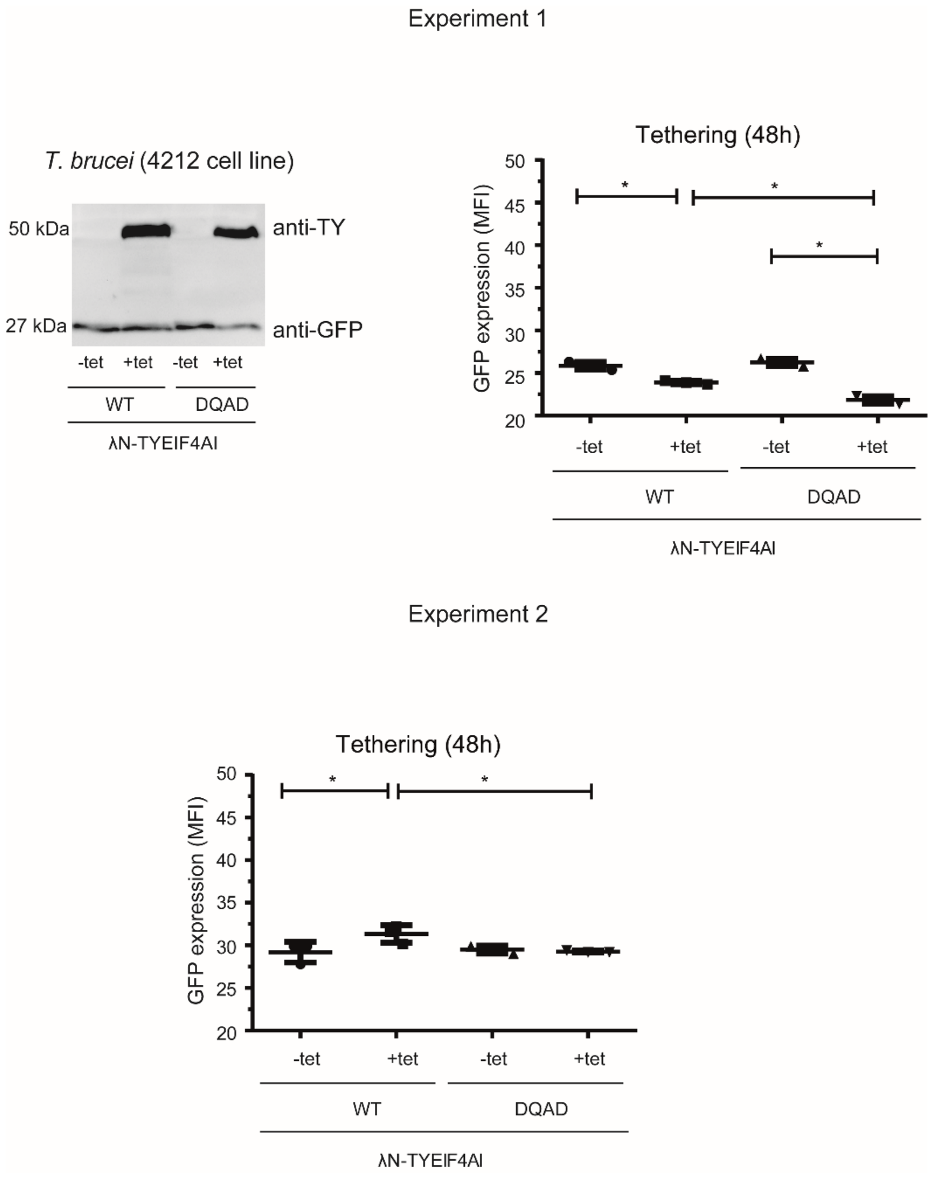

S5 Fig – Evaluation of the effect of the tethered EIF4AI, wild type and DQAD mutant, on the eGFP reporter from a control cell line. Results from two different experiments are shown, with the first experiment, shown on top, evaluated through western blot probed with the anti-TY and anti-GFP monoclonal antibodies. It confirms the expression of both the λN-TY- $EIF4AI_{WT}$ and λN-TY-$EIF4AI_{DQAD}$ in representative transgenic clones induced (+tet) or not induced (-tet) with tetracycline for 24 hours, and the effect on the eGFP expression. The same experiment was also evaluated through flow-cytometry, for a quantitative analysis of eGFP expression 48 hours after tetracycline induction. Quantitative results only for a second independent experiment are shown on the bottom. Three clones of each condition were tested for each experiment, with the results represented in the graphs as mean ± standard deviation (*= $p < 0.05$). MFI: Mean Fluorescent Intensity.
