# Supplementary material for: Distinct modes of interaction within eIF4F-like complexes and susceptibility to the RocA inhibitor for the Trypanosoma brucei EIF4AI translation initiation factor
Source: PLoS One. 2025 May 9;20(5):e0322812. doi: 10.1371/journal.pone.0322812 (PMC12063893; doi:10.1371/journal.pone.0322812)
Supplement: S6 Fig — The effect of the λN-TY-EIF4E1 expression on the eGFP reporter encoded by the mRNA with the boxB motif on its 3’UTR (from the 4213 cell line) is shown on the left. The western blots on top, probed with the anti-TY monoclonal antibody, confirm the expression of λN-TY- EIF4E1 in representative transgenic clones induced (+tet, 24 and 48h) or not induced (0h) with tetracycline, with the BiP chaperone used as loading control. The quantitative analyses of the eGFP expression 24 and 48 hours after tetracycline induction, for two independent experiments, are shown below. Equivalent experiments, shown on the right, were performed in order to assess the effect of the λN-TY-EIF4E1 expression on the eGFP reporter encoded by the control mRNA (from the 4212cell line). Three clones were tested for each experiment from each condition, with the results represented mean ± standard deviation. (PDF) [file pone.0322812.s010.pdf]

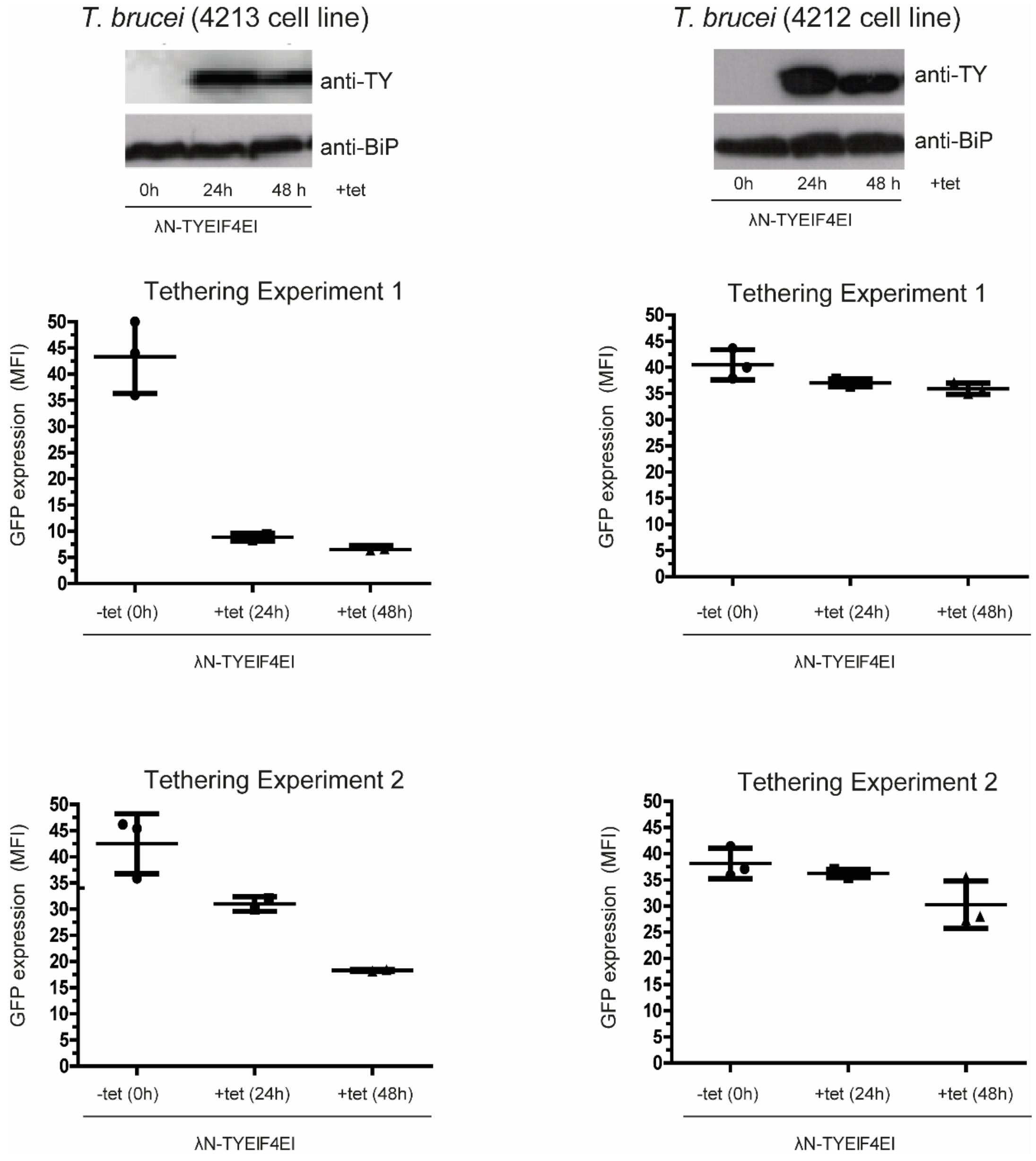


**S6 Fig – Evaluation of the tethered EIF4E1, used as a control, on the expression of the eGFP reporter mRNA.** The effect of the λN-TY-EIF4E1 expression on the eGFP reporter encoded by the mRNA with the boxB motif on its 3'UTR (from the 4213 cell line) is shown on the left. The western blots on top, probed with the anti-TY monoclonal antibody, confirm the expression of λN-TY- EIF4E1 in representative transgenic clones induced (+tet, 24 and 48h) or not induced (0h) with tetracycline, with the BiP chaperone used as loading control. The quantitative analyses of the eGFP expression 24 and 48 hours after tetracycline induction, for two independent experiments, are shown below. Equivalent experiments, shown on the right, were performed in order to assess the effect of the λN-TY-EIF4E1 expression on the eGFP reporter encoded by the control mRNA (from the 4212cell line). Three clones were tested for each experiment from each condition, with the results represented mean ± standard deviation.
