# Supplementary material for: Distinct modes of interaction within eIF4F-like complexes and susceptibility to the RocA inhibitor for the Trypanosoma brucei EIF4AI translation initiation factor
Source: PLoS One. 2025 May 9;20(5):e0322812. doi: 10.1371/journal.pone.0322812 (PMC12063893; doi:10.1371/journal.pone.0322812)
Supplement: S7 Fig — For all the experiments, each spot represents an average of cell-density values from three different growth curves set up in parallel. These values were measured 48 hours after the cells were incubated with the RocA inhibitor and were used to define the growth inhibition results shown in Figures 3A and 5B. The experiments with the T, brucei procyclic cells are shown on the left, while on the right are shown those performed with the L. infantum promastigotes. (PDF) [file pone.0322812.s011.pdf]

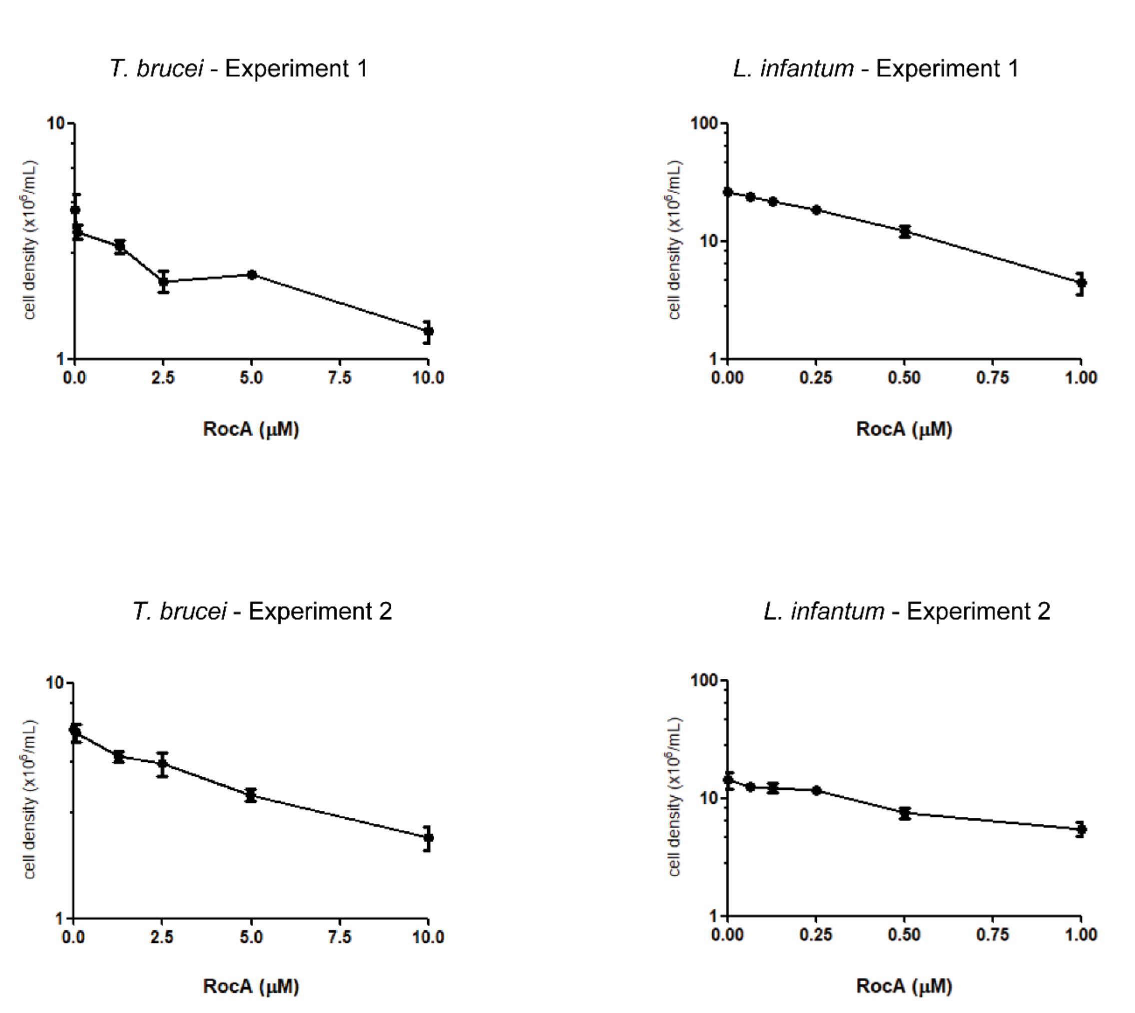

S7 Fig – Rocaglamide (RocA) effect on the growth of both *Trypanosoma brucei* and *Leishmania infantum*. For all the experiments, each spot represents an average of cell-density values from three different growth curves set up in parallel. These values were measured 48 hours after the cells were incubated with the RocA inhibitor and were used to define the growth inhibition results shown in Figures 3A and 5B. The experiments with the *T, brucei* procyclic cells are shown on the left, while on the right are shown those performed with the *L. infantum* promastigotes.
