# Supplementary material for: Distinct modes of interaction within eIF4F-like complexes and susceptibility to the RocA inhibitor for the Trypanosoma brucei EIF4AI translation initiation factor
Source: PLoS One. 2025 May 9;20(5):e0322812. doi: 10.1371/journal.pone.0322812 (PMC12063893; doi:10.1371/journal.pone.0322812)
Supplement: S8 Fig — The alignment was performed using the MAFFT software, with amino acids identical in 60% or more of the sequences highlighted in black, whilst amino acids defined as similar in 60% or more of the sequences shown in grey. (PDF) [file pone.0322812.s012.pdf]

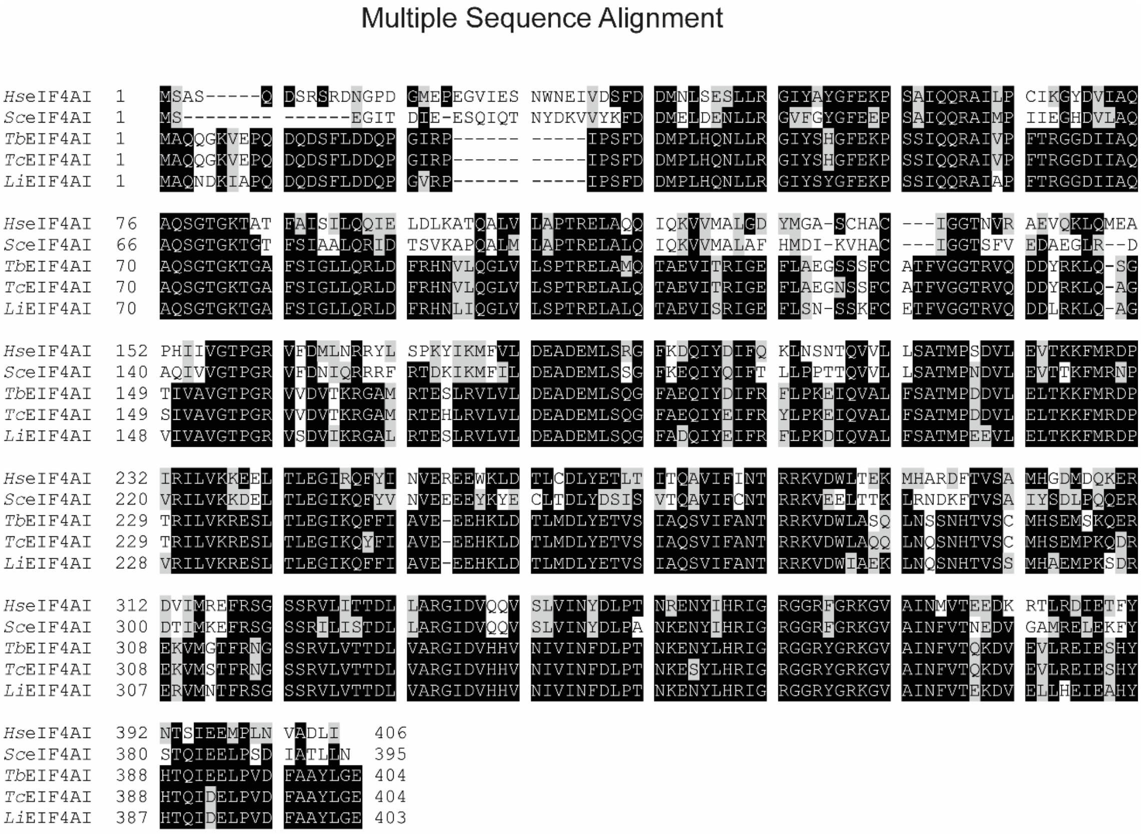

S8 Fig – Multiple sequence alignment comparing the human and yeast eIF4AI sequences with the *Leishmania* and *Trypanosoma* EIF4AI orthologues. The alignment was performed using the MAFFT software, with amino acids identical in 60% or more of the sequences highlighted in black, whilst amino acids defined as similar in 60% or more of the sequences shown in grey.
