# Supplementary material for: Distinct modes of interaction within eIF4F-like complexes and susceptibility to the RocA inhibitor for the Trypanosoma brucei EIF4AI translation initiation factor
Source: PLoS One. 2025 May 9;20(5):e0322812. doi: 10.1371/journal.pone.0322812 (PMC12063893; doi:10.1371/journal.pone.0322812)
Supplement: S9 Fig — Ramachandran plot scores for the modelled EIF4AI orthologues from T. brucei and Leishmania are shown on top. Values for all residues in allowed regions are derived from the sum of the residues in most favoured regions with those in additionally allowed and generously allowed regions. The Root Mean Square Deviation (RMSD) analysis is shown below. The RMSD is 0 for identical structures and increases with greater differences between the two structures. A RMSD near zero in the alignment between structures indicates significant structural similarity. (PDF) [file pone.0322812.s013.pdf]

Ramachandran plot score

	Residues in most favored regions	Residues in additionally allowed regions	Residues in generally allowed regions	Residues in disallowed regions	All residues in allowed regions
***T. brucei* EIF4AI**	**96.2%**	**3.6%**	**0.3%**	**0.0%**	**100%**
***L. infantum* EIF4AI**	**95.6%**	**5.4%**	**0.0%**	**0.0%**	**100%**

Root Mean Square Deviation (RMSD)

RMSD (Å)	eIF4A	*T. brucei* EIF4AI	*L. infantum* EIF4AI
**eIF4A**	**0.000**	**1.167**	**1.132**
***T. brucei* EIF4AI**		**0.000**	**0.148**
***L. infantum* EIF4AI**			**0.000**

S9 Fig – Assessment of the quality of the models generated for the interactions between the *T. brucei* and *Leishmania* EIF4AI with RNA and RocA. Ramachandran plot scores for the modelled EIF4AI orthologues from *T. brucei* and *Leishmania* are shown on top. Values for all residues in allowed regions are derived from the sum of the residues in most favoured regions with those in additionally allowed and generously allowed regions. The Root Mean Square Deviation (RMSD) analysis is shown below. The RMSD is 0 for identical structures and increases with greater differences between the two structures. A RMSD near zero in the alignment between structures indicates significant structural similarity.
