# Supplementary material for: Distinct modes of interaction within eIF4F-like complexes and susceptibility to the RocA inhibitor for the Trypanosoma brucei EIF4AI translation initiation factor
Source: PLoS One. 2025 May 9;20(5):e0322812. doi: 10.1371/journal.pone.0322812 (PMC12063893; doi:10.1371/journal.pone.0322812)
Supplement: S10 Fig — Individual maps are represented with the colour codes indicated in the figure. (PDF) [file pone.0322812.s014.pdf]

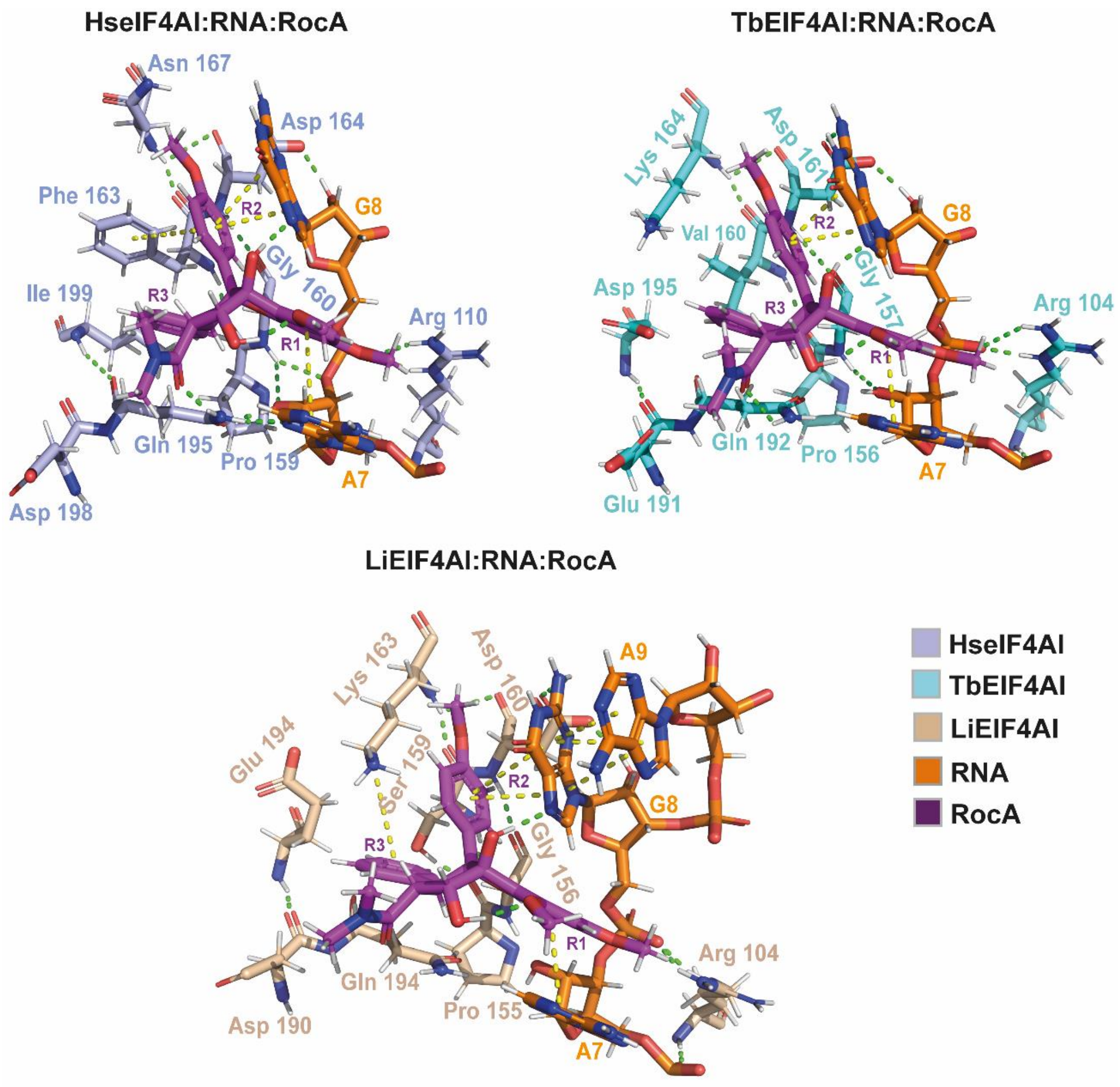


**S10 Fig – 3D maps of the RocA binding pocket, based on the structure of the human eIF4AI:RNA interacting with RocA as well as the model for the TbEIF4AI:RNA:RocA and LiEIF4AI:RNA:RocA complexes.** Individual maps are represented with the colour codes indicated in the figure.
